# Design of Polypeptides Self-Assembling into Antifouling Coatings: Exploiting Multivalency

**DOI:** 10.1021/acs.biomac.2c00170

**Published:** 2022-08-11

**Authors:** Nicolò Alvisi, Chuanbao Zheng, Meike Lokker, Victor Boekestein, Robbert de Haas, Bauke Albada, Renko de Vries

**Affiliations:** † Laboratory of Physical Chemistry and Soft Matter, Wageningen University & Research, Stippeneng 4, 6708 WE Wageningen, The Netherlands; ‡ Laboratory of Organic Chemistry, Wageningen University & Research, Stippeneng 4, 6708 WE Wageningen, The Netherlands

## Abstract

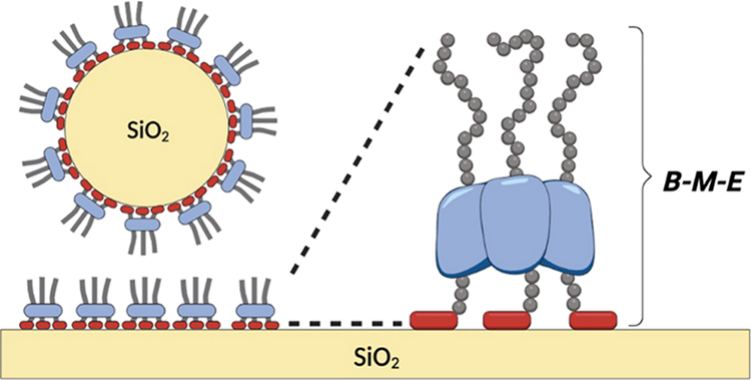

We propose to exploit multivalent binding of solid-binding peptides (SBPs) for the physical attachment of antifouling polypeptide brushes on solid surfaces. Using a silica-binding peptide as a model SBP, we find that both tandem-repeated SBPs and SBPs repeated in branched architectures implemented via a multimerization domain work very well to improve the binding strength of polypeptide brushes, as compared to earlier designs with a single SBP. At the same time, for many of the designed sequences, either the solubility or the yield of recombinant production is low. For a single design, with the domain structure ***B***-***M***-***E***, both solubility and yield of recombinant production were high. In this design, ***B*** is a silica-binding peptide, ***M*** is a highly thermostable, de novo-designed trimerization domain, and ***E*** is a hydrophilic elastin-like polypeptide. We show that the ***B***-***M***-***E*** triblock polypeptide rapidly assembles into highly stable polypeptide brushes on silica surfaces, with excellent antifouling properties against high concentrations of serum albumin. Given that SBPs attaching to a wide range of materials have been identified, the ***B***-***M***-***E*** triblock design provides a template for the development of polypeptides for coating many other materials such as metals or plastics.

## Introduction

A key challenge in designing interfaces between artificial materials and biological materials (foods, body fluids, tissues, microorganisms, etc.) is to prevent unwanted adsorption and accumulation of biological macromolecules and microorganisms at the interface.^1,2^ For solid surfaces, coatings with a hydrophilic polymer brush can prevent biofouling to a large degree.^3,4^ The brushes can be attached to the surfaces either chemically^2,5^ or via multiple weaker physical bonds,^6^ with both approaches having their own advantages and disadvantages.

The best-known examples of physical attachment of antifouling brushes are comb polymers with surface-binding main chains and antifouling side chains, such as the widely used poly(l-lysine)-*g*-poly(ethylene glycol) (PLL-*g*-PEG) graft copolymers with PLL main chains and a high density of short PEG side chains.^6,7^ This polymer is particularly effective for negatively charged surfaces such as glass.^6^

For strong physical attachment to a wider range of possibly important solid materials (metals, plastics, minerals), solid-binding peptides (SBPs) are a useful strategy.^8,9^ SBPs are short amino acid sequences selected using enrichment strategies such as phage display^10,11^ for binding strongly to certain solid surfaces. A plethora of SBPs has been developed, with binding affinity for a wide range of materials including oxides,^12,13^ metals,^14^ and plastics.^15−17^

Despite their application potential, their actual use for immobilizing molecular cargo to solids is still limited. In fact, the binding of SBPs to solids is not permanent since their dissociation constants are typically larger than 100 nM, depending on the solution conditions.

**Figure 1:**
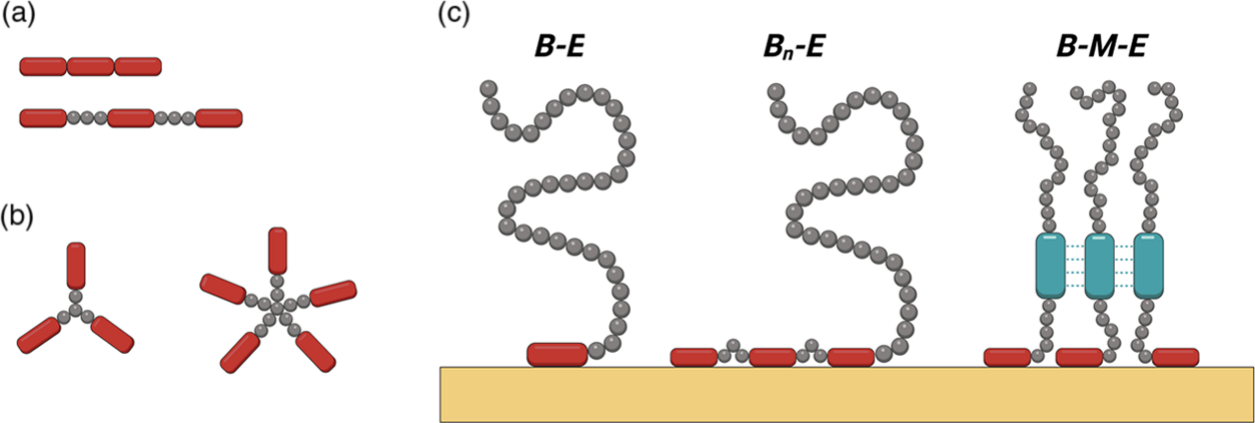
Multivalent display of solid-binding peptides (SBPs). (a) Linear tandem repeats. (b) Star-like architectures on the scaffold. (c) Brush-forming polypeptides with SBPs as “surface stickers”. Left: ***B**-**E*** diblock architecture, middle: ***B***_*n*_-***E*** diblock architecture with tandem-repeated SBPs, right: ***B***-***M***-***E*** star-like architecture with oligomerizing middle block ***M***.

An obvious strategy to engineer stronger binding using SBPs is to introduce multivalent binding, displaying multiple SBPs on a scaffold structure (Figure 1a,b).

A first approach is to use tandem-repeated SBPs, connected directly or via spacers (Figure 1a). Hassert et al.^18^ reported the development of a strongly silica-binding peptide (highSP) containing quadruple repeats of the minimal binding motif of a weaker precursor. Cho et al.^19^ showed that triplicate tandem repeats of a ZnO-binding peptide led to stronger and more stable peptide coatings on ZnO particles than the single SBP. On the other hand, Seker et al.^20^ showed that the use of multiple SBPs does not necessarily lead to stronger binding. Recent findings by Bansal et al.^21^ suggest that in the latter case, lack of improved binding was due to the fact that the tandem-repeated SBP could not simultaneously adopt the optimal spatial conformation for binding for all tandem repeats.

Among the possible scaffold architectures for multivalent display,^22^ star-like architectures have been especially well explored for SBPs (Figure 1b). Tang et al.^23^ reported a 100-fold increase in binding affinity for a tetravalent dendron exposing hydroxyapatite-binding peptides compared to the monovalent peptide. Similar results were also reported for trimeric and tetrameric dendrons with diamond-like carbon-binding peptides.^24^ Pentameric dendrons were also developed to mimic phage display.^25^ Protein-based scaffolds have also been explored. For example, Terskikh et al.^26^ exploited the pentameric assembly domain of the cartilage oligomeric matrix protein to increase the binding affinity of a synthetic peptide for cell-type specific surface recognition. Similarly, Sano et al.^27^ fused a titanium-binding aptamer to the L chain of ferritin, creating a cage architecture of 24 polypeptide chains with a 1000-fold increase in binding affinity compared to the single SBP.

Here, we focus on the physical attachment of antifouling polypeptide brushes to solid surfaces using SBPs as surface stickers, with the potential to design self-assembling brushes suitable for a wide range of solid substrates. Previously, we have reported the design of a protein-based diblock polymer ***B***-***E*** comprised of a silica-binding SBP, ***B***, and a hydrophilic elastin-like domain ***E*** (Figure 1c, left). We showed that these polymers assemble into stable hydrophilic brushes on silica surfaces and nanoparticles.^28^ While the coatings were relatively stable against prolonged rinsing with phosphate-buffered saline (PBS), solutions with higher ionic strengths (>0.5 M NaCl) were still able to displace the adsorbed polymers.

For the design of polypeptide brushes with good resistance to displacement in a wider range of solvent conditions, we here explore whether we can employ multivalent SBP binding. Based on the literature on multivalent binding of SBPs discussed above, we consider both designs ***B***_*n*_-***E*** with tandem-repeated SBPs (Figure 1c, middle) and designs ***B***-***M***-***E*** with star-like architectures, where ***M*** is an oligomerizing domain (Figure 1c, right). Unfortunately, we find that many designs do not satisfy basic prerequisites, such as simple production and purification and good solubility. However, we find that these prerequisites are satisfied by a specific ***B***-***M***-***E*** design, where ***M*** is a highly stable, de novo-designed, trimer-forming domain. For this particular design, we investigate its self-assembly on silica surfaces, the stability of the brushes it forms, and the antifouling properties of these brushes with respect to bovine serum albumin.

## Materials and Methods

### Construction of Expression Plasmids for Polypeptides

For the construction of ***B***_*n*_-***E*** diblocks with tandem-repeated binding blocks ***B*****^*RT*^**, a synthetic gene encoding for ***B***_*n*_-***E******^S^***_20_ was synthesized by Twist Bioscience. For *n* = 1, the block encodes a poly-histidine tag for downstream purification, the ***B***^***RT***^ = (RTHRK)_4_ tag, and the linker polypeptide ***E***_20_^***S***^ = (VPGSG)_20_. For *n* = 2, the block encodes a poly-histidine tag for downstream purification, two ***B***^***R***^***^T^*** = (RTHRK)_4_ tags interspaced by a linker ***E***_3_^***S***^ = (VPGSG)_3_, and the linker polypeptide ***E***_20_^***S***^ = (VPGSG)_20_. For *n* = 3, the block encodes a poly-histidine tag for downstream purification, three ***B***^***RT***^ = (RTHRK)_4_ tags interspaced by a linker ***E***_3_^***S***^ = (VPGSG)_3_, and the linker polypeptide ***E***_20_^***S***^ = (VPGSG)_20_. The synthetic fragments were designed to contain the features needed for PRe-RDL cloning, as described by McDaniel.^29^ The fragment was digested with **Bam**HI/*Acu*I and ligated into a **Bam**HI/*Acu*I-digested pET-24a(+) vector containing the ***E***_20_^***S***^ = (VPGSG)_20_ sequence.

For the construction of ***B***-***M***-***E*** triblocks, a synthetic gene encoding for the ***B***^***RT***^***-M*** block was synthesized by Integrated DNA Technologies (Leuven, Belgium). The block encodes a poly-histidine tag for downstream purification, the ***B***^***RT***^ = (RTHRK)_4_ tag, a linker polypeptide ***E***_5_^***S***^ = (VPGSG)_5_, and the multimerization domains ***M***. The synthetic fragment was designed to contain the features needed for PRe-RDL cloning, as described by McDaniel.^29^ The fragment was digested with **Bam**HI/*Acu*I and ligated into a **Bam**HI/*Acu*I-digested pET-24a(+) vector containing the ***E***_40_^***S***^ = (VPGSG)_40_ sequence.

For all constructs, plasmid DNA was transformed into*Escherichia coli* DH5α via heat shock. Colonies containing the correct DNA insert were selected and confirmed by DNA sequencing. Then, the plasmid was transformed into*E. coli* T7-Express. The full DNA and amino acid sequences of the polypeptides are reported in Tables S1 and S2.

### Protein Expression

*E. coli* T7-Express containing the expression plasmids for the polypeptides was cultured at 37 °C/215 rpm for 16 h in 25 mL terrific broth (TB) medium containing 50 μg/mL kanamycin. The starter culture was inoculated in 1 L of lysogeny broth (LB) medium containing 50 μg/mL kanamycin and incubated at 37 °C/215 rpm until OD_600_ > 0.6. When necessary, 0.5% d-glucose was added. Then, isopropylthio-β-galactoside (IPTG) was added to a final concentration of 1 mM and cells were incubated overnight at 18 °C/215 rpm before harvesting.

### Protein Purification

Bacterial cells were centrifuged at 6000 rpm for 30 min at 4 °C and resuspended in 30 mL of cold extraction buffer (50 mM Tris pH 8.00, 300 mM NaCl, 30 mM imidazole). Phenylmethylsulfonyl fluoride (PMSF) was added to the cell suspension to a final concentration of 1 mM. Cells were lysed via sonication (Q125 Sonicator, QSonica). The cell lysate was centrifuged for 30 min at 30,000*g* at 4 °C to pellet the insoluble fraction. Next, the polypeptide was purified using immobilized metal ion affinity chromatography (IMAC). The sample was injected in an IMAC column (Bio-Scale Mini Profinity IMAC cartridge, Bio-Rad Laboratories) and washed with extraction buffer containing 2 M NaCl to remove DNA contamination. The polypeptide was eluted with a linear gradient from extraction buffer to elution buffer (50 mM Tris pH 8.00, 300 mM NaCl, 300 mM imidazole). The purity of the polypeptides was assessed by sodium dodecyl sulfate-polyacrylamide gel electrophoresis (SDS-PAGE).

### Matrix-Assisted Laser Desorption/Ionization-Time of Flight (MALDI-TOF) Analysis

To confirm the size of the polypeptide, its molecular weight was determined using matrix-assisted laser desorption/ionization (MALDI) mass spectrometry. The spectrum was obtained using a Bruker UltraFlextreme machine. The sample was prepared following the instructions provided by the manufacturer.

### Circular Dichroism

For circular dichroism measurements, a Jasco Spectropolarimeter J-715 was used. Data was collected and analyzed with Jasco software. For the sample preparation, the protein was dissolved at a concentration of 0.1 mg/mL in phosphate-buffered saline (PBS) pH. 7.4. The solution was sonicated for 10 min to reduce the presence of aggregates before the measurement. A quartz cuvette with a 1 mm path was used. For the spectrum measurements, the instrument was set to continuous scanning mode, with a data pitch of 0.1 nm, a scanning speed of 50 nm/min, and a band width of 2 nm. Each spectrum was accumulated 20 times. For the temperature ramp, the ellipticity was measured at 222 nm while increasing the temperature of the sample from 20 to 95 °C, at 1 °C/min.

### Dynamic Light Scattering

For dynamic light scattering measurements, a ZS-Nano (Malvern, U.K.) instrument was used. Light scattering was measured at a scattering angle of 173°, at *T* = 20 °C. The reported hydrodynamic sizes were obtained using the Zetasizer software version 7.13 (Malvern, U.K.). Nonfunctionalized silica microspheres with a diameter of 163 nm were purchased from Bangs Laboratories. For dynamic light scattering (DLS) on ***B***-***M***-***E*** solutions, the protein was dissolved in the appropriate buffer (phosphate buffer with increasing concentrations of NaCl). The solutions were filtered using a 0.22 μm filter and sonicated for 10 min to reduce the presence of aggregates before the measurement. For each sample, continuous measurements were performed, with the duration of each measurement defined by the instrument. Each reported hydrodynamic size is the average of 10 measurements. For measurements on silica nanoparticles coated with ***B***_*n*_-***E*** or ***B***-***M***-***E***, the polypeptide solution and the silica nanoparticles (0.01% v/v) were sonicated for 10 min. The polypeptide solution was filtered using a 0.22 μm filter. Increasing concentrations of protein were incubated with the nanoparticle solution for 10 min. For each sample, continuous measurements were done, each reported hydrodynamic size is the average of 10 measurements, with the duration of the measurement defined by the instrument. For the ζ potential measurements, the samples were prepared following the same procedure. A dip cell (Malvern, U.K.) was used. Each reported ζ potential value is the average of 15 measurements.

### Quartz Crystal Microbalance with Dissipation Monitoring (QCM-D)

A QSense E4 QCM-D instrument (Biolin Scientific, Sweden) was used to quantify polypeptide binding to silica. QCM sensors coated with SiO_2_ were obtained from Biolin Scientific and cleaned according to the provided instructions. For the salt dependency measurements, each sensor was equilibrated with the appropriate buffer (PB, PBS 150 mM NaCl, PBS + 0.5 M NaCl, PBS + 1 M NaCl) at a flow rate of 50 μL/min until a stable baseline was reached. ***B***_*n*_-***E*** or ***B***-***M***-***E*** were dissolved to a final concentration of 10 μM and dialyzed in the previously mentioned buffers. The protein solutions were sonicated for 10 min prior to the measurement. Analysis of QCM-D data was performed using QSense software (Biolin Scientific, Sweden). For the antifouling test, each sensor was equilibrated with PBS at a flow rate of 50 μL/min until a stable baseline was reached. The SiO_2_ sensors were coated with PLL-PEG (1 mg/mL), the diblock polypeptide ***B***-***E*** (5 μM), and ***B***-***M***-***E*** (5 μM) until a stable value of frequency shift (Δ*f* ) was reached. Coated and uncoated sensors were then flushed with a solution of bovine serum albumin (2 mg/mL). Analysis of QCM-D data was performed using QSense software.

### Atomic Force Microscopy (AFM) Imaging in Air

Atomically flat silica surfaces were obtained using silicon wafers (Siltronic AG) with a 2–3 nm oxide layer due to natural oxidation with oxygen in air. Silica surfaces were cleaned with MilliQ water and ethanol and plasma cleaned for 5 min. For AFM imaging, 30 μL of the protein solution was deposited on a cleaned silica surface and incubated for 1 min. After that, samples were gently rinsed with MilliQ water and carefully dried with nitrogen. Samples were imaged with a Multimode Bruker AFM (Bruker, California) using the automatic ScanAsyst imaging mode. ScanAsyst air tips were used with a nominal radius < 10 nm.

## Results and Discussion

In our previous work, we found that ***B***-***E*** diblocks with silica-binding blocks ***B***^***RT***^ = (RTHRK)_4_^18^ and elastin-like hydrophilic blocks ***E*****^*S*^**_40_ = (VPGSG)_40_ satisfy the prerequisites of simple production and purification as well as good solubility and silica binding.^28^ Therefore, our current designs still use ***B*****^*RT*^** as binding blocks for silica surfaces and ***E*****^*S*^**_40_ as hydrophilic brush-forming blocks. We designed both ***B***_*n*_-***E*** diblocks with tandem-repeated binding blocks ***B*****^*RT*^** (up to three repeats) and ***B***-***M***-***E*** triblocks, with oligomerizing midblocks ***M*** of different valences *m*. As oligomerizing blocks ***M***, we selected four naturally occurring^30−33^ and three highly stable de novo computationally designed oligomerizing domains.^34^ In some cases, the N-terminal of the oligomerization blocks did not allow for suitable display of the binding block. For these cases, we used ***M***-***B***-***E*** triblock designs instead. Finally, we used short ***E*****^*S*^**_*n*_ sequences (*n* = 3–5) as linkers for connecting oligomerization and binding blocks and for connecting binding blocks to form tandem repeats. The designs are listed in Table 1. They all feature N-terminal His-tags, were recombinantly produced in *E. coli*, and purified using IMAC affinity chromatography. For full DNA and protein sequences, see Tables S1 and S2.

**Table 1:** Overview of Tested Designs

oligomerization block M			protein design	
name	origin	*m*	type	sequence
			***B***_*n*_-***E***	***B******^RT^***-***E******^S^***_40_
			***B***_*n*_-***E***	***B^RT^***-***E^S^***_3_-***B******^RT^***-***E^S^***_40_
			***B***_*n*_-***E***	***B******^RT^***-***E^S^***_3_-***B******^RT^***-***E^S^***_3_-***B******^RT^***-***E^S^***_40_
foldon^30^	natural	3	***B***-***M***-***E***	***B******^RT^***-***M******^foldon^***-***E^S^***_40_
I53.50A^33^		3	***B***-***M***-***E***	***B******^RT^***-***E^S^***_5_-***M******^I53.50A^***-***E^S^***_40_
LSM-α^31^		7	***B***-***M***-***E***	***B******^RT^***-***E^S^***_3_-***M******^LSM-α^***-***E^S^***_40_
TRAP^32^		11	***M***-***B***-***E***	***M******^TRAP^***-***E******^S^***_3_-***B******^RT^***-***E******^S^***_40_
HR00C3_2^34^	de novo	3	***B***-***M***-***E***	***B******^RT^***-***E^S^***_3_-***M*****^*HR00C3_2*^**-***E^S^***_40_
HR00C3_2^34^		3	***B***-***M***	***B******^RT^***-***E^S^***_3_-***M******^HR00C3_2^***
ank1C4_2^34^		4	***M***-***B***-***E***	***M******^ank1C4_2^***-***E^S^***_3_-***B******^RT^***-***E^S^***_40_
1na0C3_3^34^		3	***M***-***B***-***E***	***M******^1na0C3_3^***-***E^S^***_3_-***B******^RT^***-***E^S^***_40_

SDS-PAGE analysis of the expression and purification of the tandem-repeat designs ***B***_*n*_-***E*** (*n* = 1–3) is shown in Figure S1. We found that the expression of the tandem-repeat designs decreases rapidly with increasing *n*. Some improvement in expression for *n* = 2 and 3 was found using LB medium containing 0.5% d-glucose (Figure S1b–f), suggesting that the expression at higher *n* is limited by the toxicity of the polypeptide.^35^ QCM-D analysis of brush formation by ***B***_*n*_-***E*** diblocks with *n* = 1, 2 on silica-coated quartz sensors showed that the tandem-repeat strategy is effective in improving the brush stability against displacement by high ionic strength solutions (Figure S2). Nonetheless, the tandem-repeat designs will not be further explored because of the problems in the production of these polymers. Instead, we will focus on employing oligomerizing midblocks ***M*** with star-like display of the SBPs (Figure 1c, right).

While all designs containing natural oligomerization domains could be expressed (Figure S3), the expression of the constructs with de novo-designed oligomerization domains was found to be higher (Figure S3e,f). However, preliminary investigations showed that the proteins with the de novo-designed oligomerization domains 1na0C3_3 and ank1C4_2 had lower solubility than the construct with the HR00C3_2 oligomerization domain. Therefore, we focus our attention on the design ***B*****^*RT*^**-***E^S^***_3_-***M^HR00C3_2^***-***E^S^***_40_, containing the trimerization domain HR00C3_2 (PDB 5K7V) designed by Fallas et al.^34^ We will subsequently refer to this construct as ***B***-***M***-***E***, with the implicit understanding that the binding block is ***B*** = ***B*****^*RT*^**-***E^S^***_3_, the multimerization block is ***M*** = ***M^HR0032_2^***, and the hydrophilic random coil block is ***E*** = ***E^S^***_40_.

**Figure 2:**
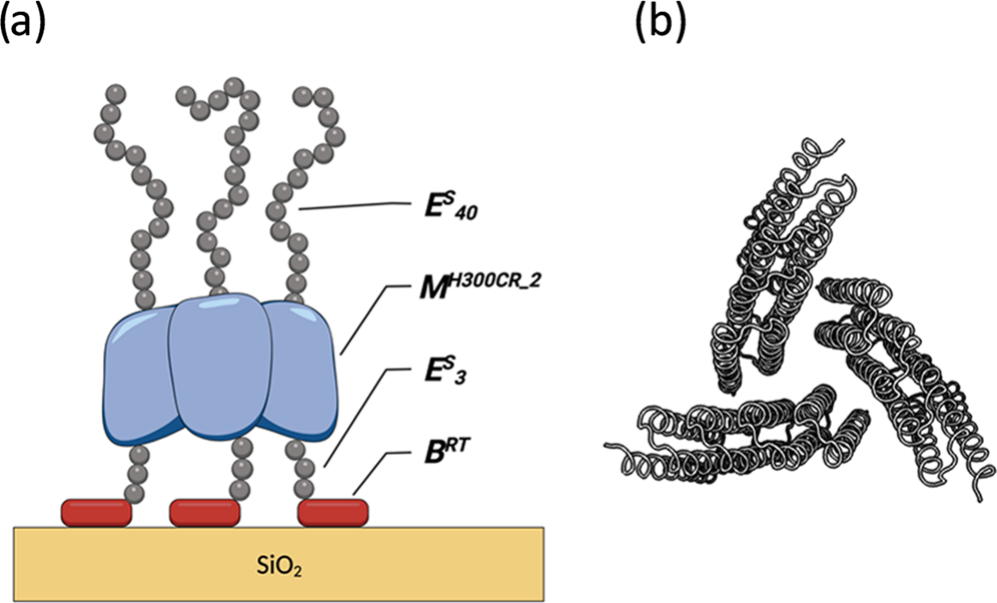
(a) Schematic representation of the structure of a trimer of the ***B***-***M***-***E*** triblock, with ***B = B*****^*RT*^***-**E^S^***_3_, ***M*** = ***M^HR00C3_2^***, and ***E**=**E^S^***_40_, adsorbed to a silica surface. (b) Crystal structure of the trimer of ***M^HR00C3_2^*** (PDB 5K7V), corresponding to a view from the top (C-terminal side) for the adsorbed ***B***-***M***-***E*** trimer.

**Figure 3:**
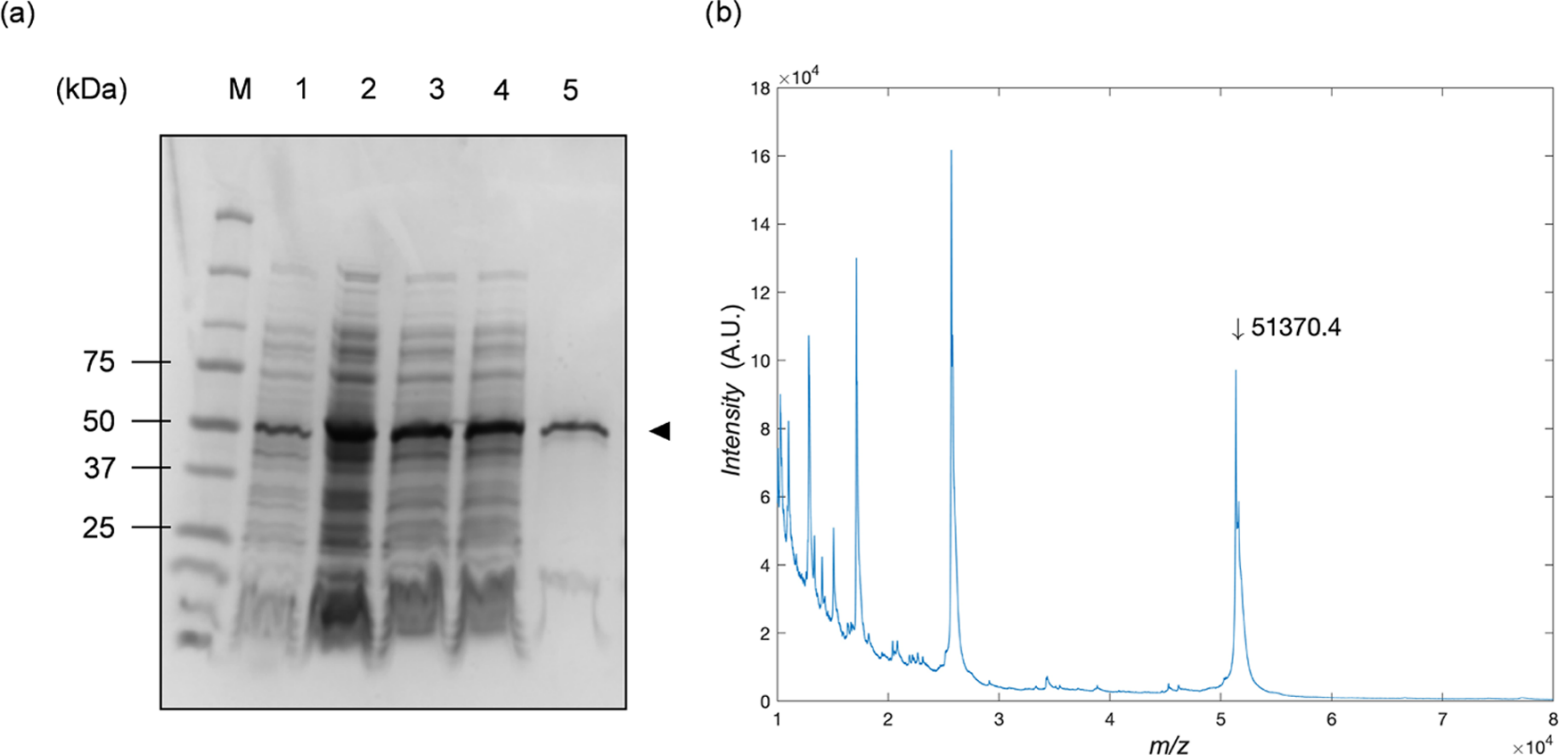
Purification and characterization of ***B***-***M***-***E*** triblock. (a) SDS-PAGE analysis. Lane 1: intact cells; lane 2: cell lysate; lane 3: soluble lysate; lane 4: IMAC flow-through; lane 5: IMAC fractions; lane M: molecular marker. (b) MALDI-TOF spectrum.

A schematic representation of the ***B***-***M***-***E*** triblocks adhering to a silica surface is shown in Figure 2a, with Figure 2b showing the experimental crystal structure of the trimer of ***M***.^34^ Representative SDS-PAGE results for the purification process and MALDI-TOF results for the purified triblock are shown in Figure 3. Although a fraction of ***B***-***M***-***E*** did not bind to the IMAC column, the protein could be eluted with high purity (Figure 3a). To confirm the correct size of the polypeptide, the purified protein was analyzed using MALDI-TOF mass spectrometry (Figure 3b). We found that the experimentally determined mass (51.370 kg/mol) is equal to the theoretically expected value (51.363 kg/mol) within the error of the measurement.

**Figure 4:**
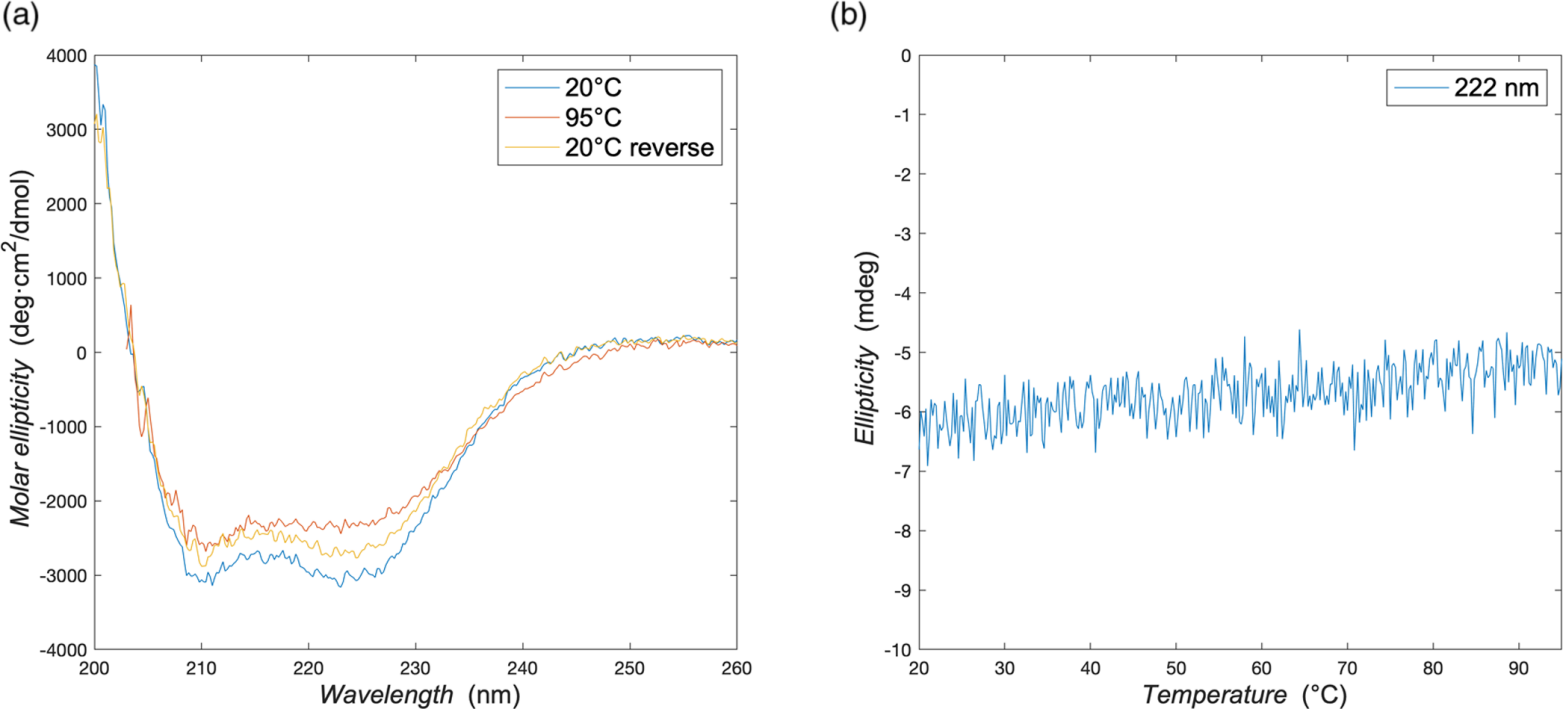
Analysis of the secondary structure of the ***B***-***M***-***E*** triblock with circular dichroism spectroscopy. (a) Molar ellipticity vs wavelength. Spectra were recorded at 20, 95, and again at 20 °C. Solution conditions: 0.1 mg/mL of protein in PBS pH 7.4. Data were collected in a cell with a 1 mm path length. (b) Ellipticity at 222 nm plotted as a function of temperature during a heating ramp (1 °C/min).

First, we investigated whether the trimerization domain ***M*** still folds correctly when included in the ***B**-**M**-**E*** triblock. The domain is extremely stable in solution, both at a high temperature and in the presence of denaturants.^34^ Furthermore, the secondary structure of the ***M*** domain is exclusively α-helical (Figure 2b), even though a large portion of the ***B**-**M**-**E*** triblock has a disordered structure. For this reason, if the circular dichroism spectrum of the ***B**-**M**-**E*** triblock shows features that are distinctive for α-helical proteins, we can assume that the trimerization domain ***M*** is correctly folded. CD spectra for the triblock are shown in Figure 4a. At room temperature (20 °C), the spectrum clearly shows two negative bands at 222 and 210 nm, characteristic of α-helical proteins.^36^ We conclude that the trimerization domain ***M*** is still correctly folded when included in the ***B**-**M**-**E*** triblock.

Next, we investigated the thermal stability of the trimerization domain in the ***B**-**M**-**E*** triblock. As shown in Figure 4a, heating the protein solution to 95 °C does not drastically affect the features of the spectrum. After cooling back to 20 °C, the spectrum is similar to the measurement before heating, indicating a fully reversible behavior and no significant unfolding. The upward shift could be caused by the partial evaporation of the sample during the heating phase, with a consequent increase in the protein concentration. As shown in Figure 4b, the measured ellipticity at 222 nm increases slightly and monotonically during a temperature ramp from 20 to 95 °C, clearly indicating that there is no unfolding before 95 °C within the timescale of the measurement.

Possibly, trimerization of the ***M*** block could be affected by the presence of the binding block ***B*** at the N-terminus or the random coil block ***M*** at the C-terminus. To investigate this, we first evaluated if the presence of the binding blocks affects trimerization. To this end, we expressed and purified the diblock ***B***-***M*** (Figure S4). We found that this polypeptide quickly aggregated in solution after purification. Since the ***M*** block, ***B*** block, and ***B***-***M***-***E*** triblocks are soluble in PBS pH 7.4, we conclude that the ***B*** and ***M*** blocks coprecipitate in our ***B***-***M*** diblock design. We hypothesize that since the binding block ***B*** is highly cationic (+12) and the trimerization domain ***M*** has a high net negative charge (−24), the observed coprecipitation is very likely caused by strong electrostatic interactions between ***B*** and ***M*** blocks (see schematic in Figure S5c).

Next, we explored the consequences of electrostatic interactions between ***B*** and ***M*** blocks for the self-assembly in solution of the ***B***-***M***-***E*** triblocks. On the one hand, we anticipate that the long hydrophilic random coil blocks ***E*** solubilize the ***B***-***M*** precipitates, in line with previous observations regarding the use of hydrophilic ELPs for the stabilization and solubilization of macromolecules.^37^ On the other hand, the ***E*** blocks may not be able to completely prevent association of ***B***-***M***-***E*** trimers in solution.

To establish solution sizes for ***B***-***M***-***E***, we used dynamic light scattering (DLS). Since we hypothesize that ***B***-***M*** interactions are electrostatically driven, we varied the salt concentration to establish whether they can be screened by the addition of salt.

**Figure 5:**
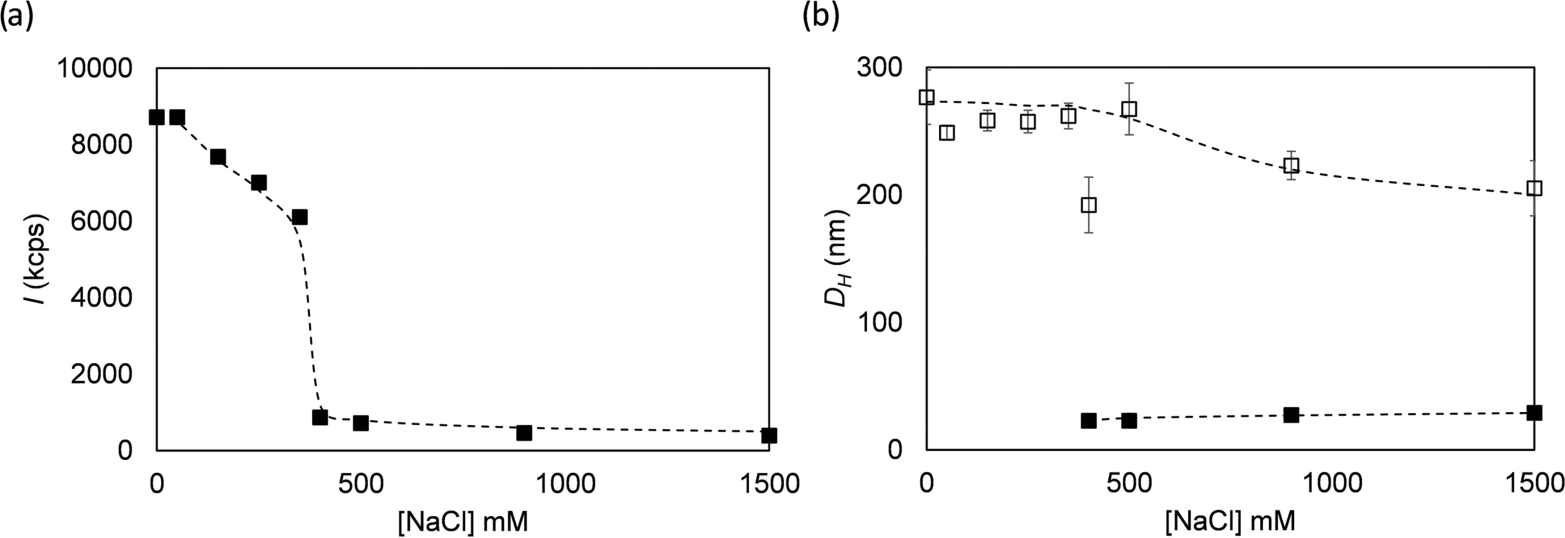
Salt dependence of self-assembly of ***B***-***M***-***E*** triblocks in solution. (a) Light scattering intensity (scattering angle 173°) vs salt concentration (concentration of NaCl added to PB, [NaCl] mM). (b) Distribution analysis of dynamic light scattering data: open symbols: hydrodynamic diameter *D*_H,eff_ (nm) for major peak, closed symbols: hydrodynamic diameter *D*_H,eff_ (nm) for minor peak. Solution conditions: 1 mg/mL protein, dissolved in 10 mM phosphate buffer (PB pH 7.4) with the indicated amount of NaCl. Dashed lines are guides to the eye.

As shown in Figure 5a, we indeed find that the scattering intensity for ***B***-***M***-***E*** solutions is highly salt-dependent, showing a sharp decrease beyond a salt concentration [NaCl] ≈ 400 mM. As shown in Figure 5b, below [NaCl] ≈ 400 mM, using a distribution analysis of the autocorrelation function, we only detected assemblies with hydrodynamic diameters *D*_H_ ≈ 250 nm. Above [NaCl] ≈ 400 mM, we observed a slight decrease of the size of the large assemblies and additionally observed assemblies with a much smaller hydrodynamic diameter *D*_H_ ≈ 22 nm (at [NaCl] = 400 mM). For [NaCl] > 400 mM, the scattering intensities corresponding to the large and small assemblies are roughly equal. Given the strong dependence of scattering intensity on particle size, this implies that at least at [NaCl] > 400 mM, the majority of the ***B***-***M***-***E*** proteins is part of the small assemblies.

A trimer of the ***M*** block has a height of 5 nm and a diameter of 7 nm. The hydrodynamic diameter of a single ***E*** block^28^ is 9 nm. Hence, the observed assemblies with a hydrodynamic diameter *D*_H_ ≈ 22 nm could very well correspond to the ***B***-***M***-***E*** trimers, but this cannot be established with certainty from the DLS measurements alone. We have attempted to separate the putative trimers from the larger assemblies using SEC at a salt concentration [NaCl] = 500 mM to establish their precise solution stoichiometry but found that the proteins eluted as a single rather broad peak covering a range of hydrodynamic sizes (Figure S5a). At the same time, SDS-PAGE indicated that peak fractions only consisted of the ***B**-**M**-**E*** protein (Figure S5b). This most likely indicates the trimers are in fast equilibrium with the larger assemblies. Indeed, all SEC fractions showed the presence of both the larger and the smaller assemblies in DLS.

**Figure 6:**
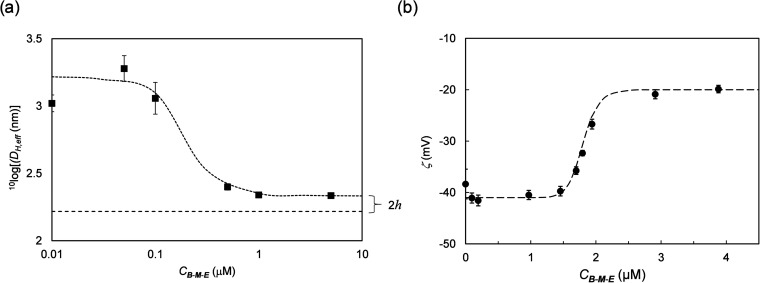
Interaction of ***B***-***M***-***E*** triblocks with 163 nm silica particles. (a) Effective hydrodynamic radius *D*_H,eff_ (nm) of the particles as determined using dynamic light scattering, vs concentration of added protein *C*_***B***-***M***-***E***_ (μM). Long-dashed line represents the diameter (*D*_H_ = 163 nm) of bare silica particles and short-dashed line is a guide for the eye. Solution conditions: silica particles were dissolved in 10 mM PB pH 7.4 at a concentration of 0.01% v/v. The protein was dissolved in 10 mM PB pH 7.4. (b) Zeta potential ζ (mV) of the particles vs the concentration of the added protein *C*_***B***-***M***-***E***_ (μM). Solution conditions: silica particles were dissolved in 10 mM PB pH 7.4 at a concentration of 0.01% v/v. The protein was dissolved in 10 mM PB pH 7.4.

The interaction of the ***B***-***M***-***E*** triblocks with silica surfaces was investigated using monodisperse nonporous silica nanoparticles with a hydrodynamic diameter *D*_H_ =163 nm. As we have shown for ***B***-***E*** diblocks, this method allows for a straightforward determination of both the concentration of protein required for a stable adsorbed polypeptide brush and the brush height via dynamic light scattering (DLS).^28^ The results are shown in Figure 6a. The results show that a protein concentration of ∼1 μM is required for the formation of a stable brush. At lower concentrations, bridging interactions lead to particle aggregation, resulting in very high scattering intensities and large effective particle radii. At higher concentrations, the bridging interactions disappear, and we measured a particle diameter of *D*_H_ = 220 nm, leading to an estimated brush height *h* ≈ 28 nm.

Electrophoretic light scattering was used to determine the change in ζ-potential of the silica particles coated with the ***B***-***M***-***E*** triblocks. The results are shown in Figure 6b. Consistently with the DLS results, we found that a concentration *C*_***B***-***M***-***E***_ of a few μM is required to give rise to a significant change of the ζ-potential, due to the self-assembly of a polypeptide brush on the surface of the silica particles. The coating makes the ζ-potential less negative, increasing from around −40 to around −20 mV.

**Figure 7:**
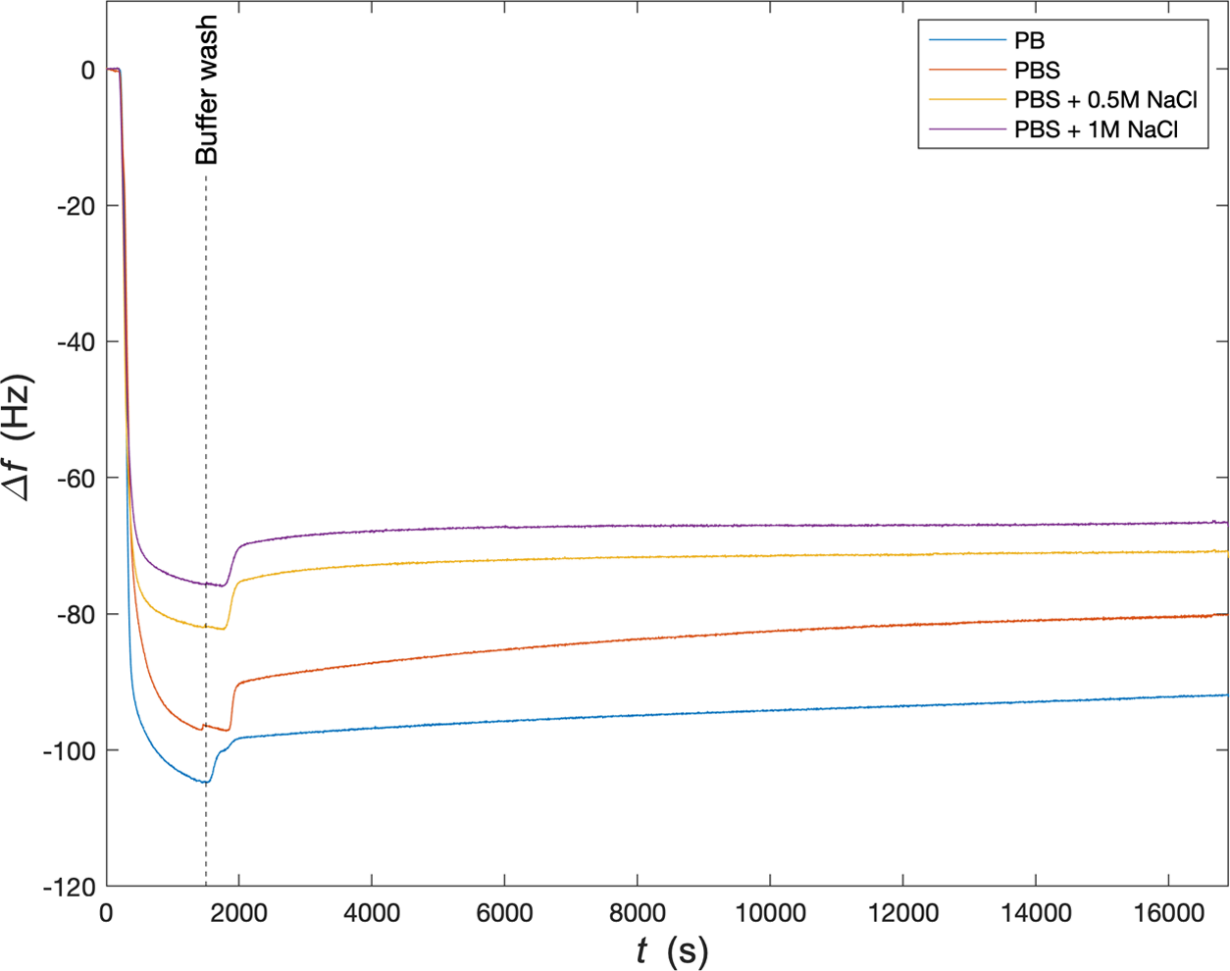
Salt dependence of brush formation on silica for 10 μM ***B***-***M***-***E*** measured with quartz crystal microbalance. Frequency shift Δ*f* (Hz) vs time *t* (s) after the start of injection of 10 μM ***B***-***M***-***E***. Buffers used for brush formation and consecutive rinsing are the following: phosphate buffer (PB, blue), phosphate-buffered saline (PBS, 150 mM NaCl, orange), PBS + 0.5 M NaCl (yellow), and PBS + 1 M NaCl (purple). Rinsing with buffer starts at the change in QCM signal following the vertical dashed line.

In our previous work on ***B***-***E*** diblocks,^28^ we found that binding with a single solid-binding peptide is not strong enough to resist displacement at higher salt concentrations, and this was part of the motivation to investigate multivalent SBP binding.We used a quartz crystal microbalance to investigate whether the ***B***-***M***-***E*** triblocks can still form stable brushes on silica surfaces at high concentrations of NaCl. The triblock polypeptide was dissolved at a concentration of 10 μM in phosphate buffer (PB), phosphate-buffered saline (PBS), PBS with 0.5 M NaCl, and PBS with 1 M NaCl. The kinetics of adsorption of the ***B***-***M***-***E*** triblocks at these different salt concentrations on silica was followed using the quartz crystal microbalance. Polypeptide solutions were flushed over the silica sensors and after a stable layer had formed, sensors were rinsed with various buffers used for the layer formation. The results are shown in Figure 7. As expected, a lower ionic strength of the buffer leads to a stronger QCM signal, but the influence of ionic strength is not very large: even at 1 M NaCl, the QCM signal at saturation for PBS + 1 M NaCl is still about 70% of that for the PB. In addition, prolonged rinsing with PBS + 1 M NaCl does not remove the layers formed from PBS + 1 M NaCl.

**Figure 8:**
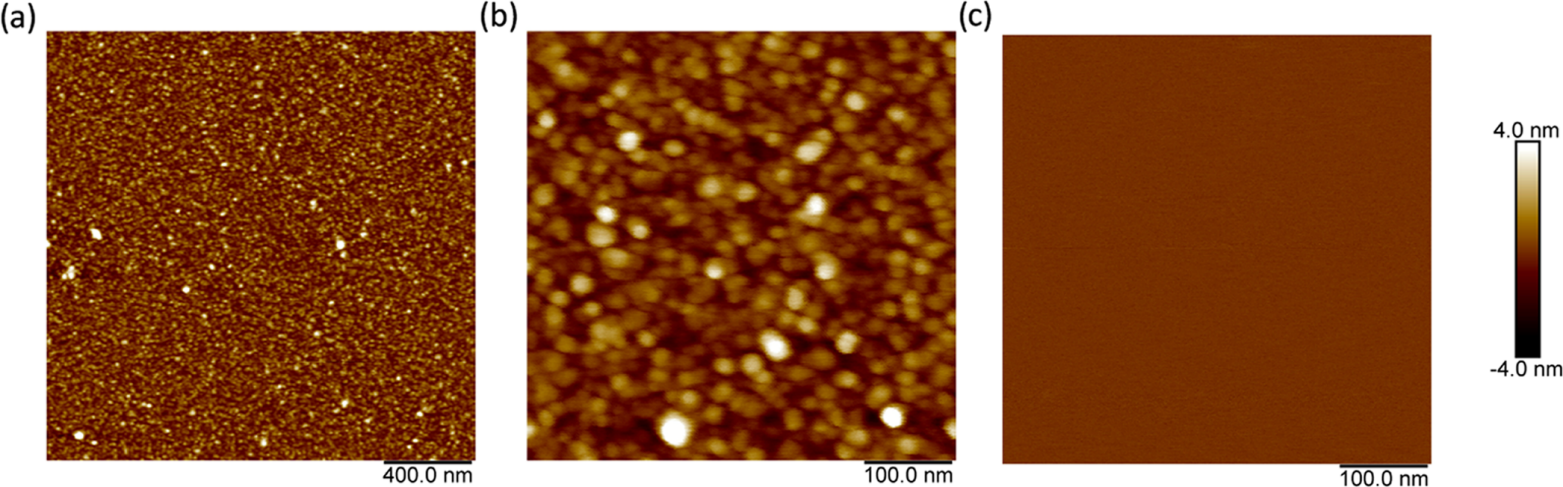
Surface morphology of ***B***-***M***-***E***-coated silica from atomic force microscopy (AFM) in air. Silica surfaces were coated for 1 min with solutions of 2 mg/mL of ***B***-***M***-***E*** in PBS, rinsed, dried, and imaged in air. (a) 2 μm × 2 μm, (b) 0.5 μm × 0.5 μm, and (c) 0.5 μm × 0.5 μm silica only, control.

According to the QCM measurements, the ***B***-***M***-***E*** trimers form stable layers on silica surfaces, with good resistance against rinsing with both low and high salt buffers. This observation, however, does not necessarily imply that the proteins adsorb as expected, with their binding domains ***B*** facing the silica and the elastin-like blocks ***E*** facing the solution. One possibility is that the larger assemblies observed in DLS for bulk solutions adsorb on the silica and form rather inhomogeneous layers. To exclude this possibility, we performed atomic force microscopy (AFM) of ***B***-***M***-***E*** layers adsorbed on silica. Imaging was done in air; the results are shown in Figure 8. We found that after drying, ***B***-***M***-***E*** layers are very thin (<10 nm) and very homogeneous over large areas of 2 μm × 2 μm. The surface morphology of the ***B***-***M***-***E*** layers as observed in AFM is that of densely packed dots, which we tentatively identify with the trimers. Hence, AFM rules out the possibility that the larger dynamic assemblies observed using DLS in solution lead to inhomogeneous coatings.

**Figure 9:**
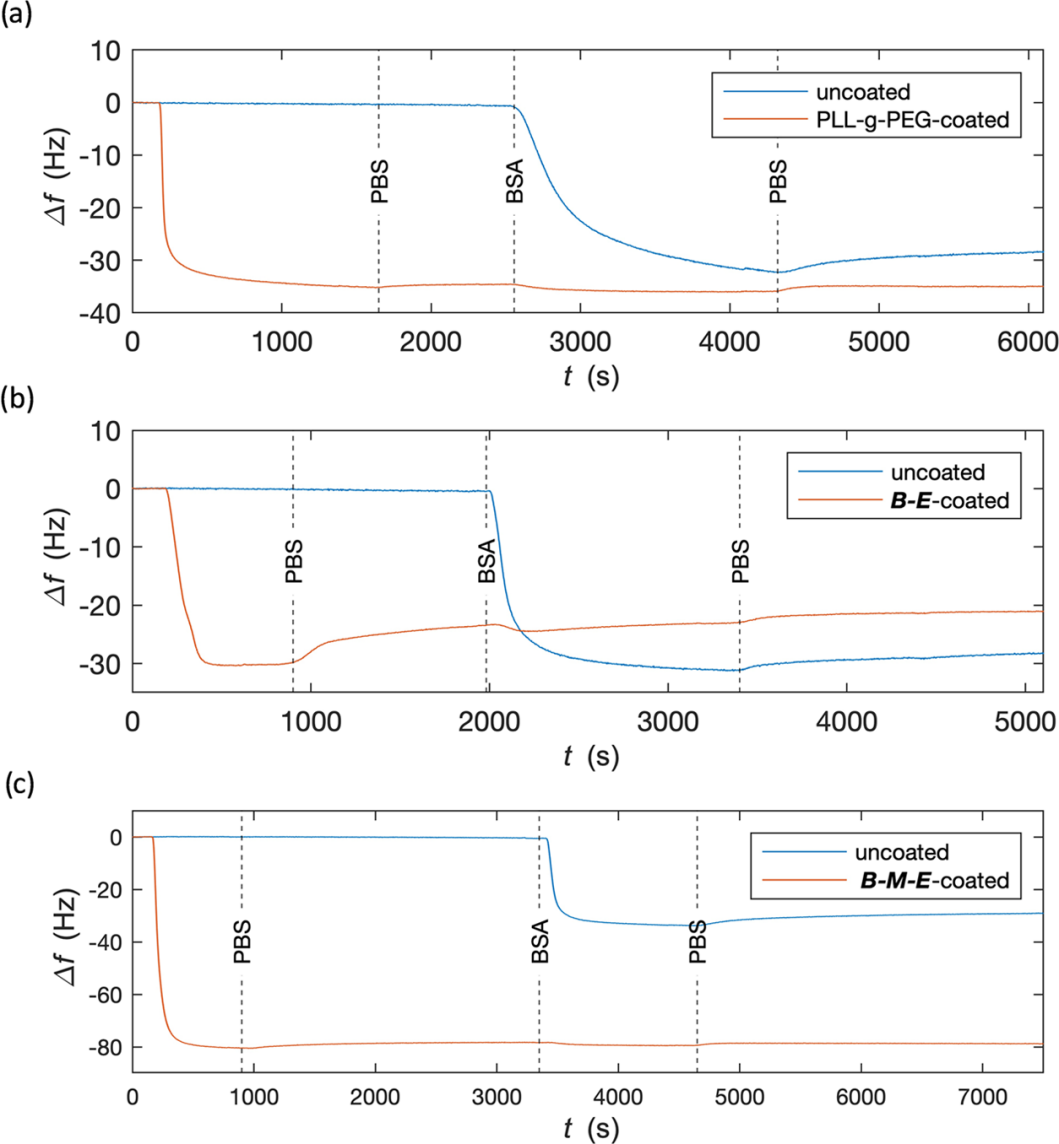
Antifouling properties of PLL-*g*-PEG, ***B***-***E***, and ***B***-***M***-***E*** polymer brushes adsorbed on the silica surface. QCM frequency shift Δ*f* (Hz) vs time *t* (s) after starting the injection of the brush-forming polymer. At the first dashed vertical lines, we switch to rinsing with PBS, at the second vertical dashed lines we switch to injecting 2 mg/mL BSA in PBS, and at the third dashed vertical lines we switch back to rinsing with PBS. Blue lines represent bare silica negative control and orange lines represent preadsorbed polymer brush. (a) PLL-*g*-PEG brush, (b) ***B***-***E*** brush, and (c) ***B***-***M***-***E*** brush.

Next to brush stability, the prevention of biological fouling is a key property for the successful application of adsorbed brushes. As a first step, we tested the antifouling ability against high concentrations of serum albumin in PBS pH 7.4. We used PLL-*g*-PEG polymers as a positive control for self-assembled antifouling brushes, and we also tested our previously designed ***B***-***E*** diblock polypeptides. QCM was used to follow the kinetics of brush assembly on silica-coated quartz sensors. After brush formation, a high concentration of bovine serum albumin (BSA, 2 mg/mL) was flushed on the sensor, followed by rinsing with PBS. As a negative control, we used a bare silica sensor. QCM results are shown in Figure 9.

For the negative control on bare silica, a strong QCM signal is observed upon injecting BSA. Only a small part of the adsorbed BSA is removed in the PBS rinsing step that follows, demonstrating the BSA has adsorbed irreversibly. The results for the PLL-*g*-PEG-coated silica sensors are as expected: the self-assembled PLL-*g*-PEG brushes effectively prevent the adsorption of BSA on the silica surface (Figure 9a). During BSA injection, a small decrease in frequency shift is recorded, indicating a small amount of BSA adsorption on the PEG tails. However, these are weakly bound and are removed again in the subsequent rinsing step.

The results for silica sensors coated with the ***B***-***E*** diblock show that brushes are gradually rinsed off by PBS (Figure 9b). Upon injecting BSA, only a very small amount of BSA adsorbs, which is again removed during the subsequent rinsing step. However, a fair amount of brush desorption takes place during the BSA injection step. The ***B***-***E*** brushes are considerably less stable than the PLL-*g*-PEG brushes with respect to rinsing with both PBS and BSA in PBS.

Finally, the results for sensors coated with the ***B***-***M***-***E*** triblock are shown in Figure 9c. At least for this assay, this polymer seems to perform better in terms of both stability and antifouling properties: changing from PBS to 2 mg/mL BSA in PBS hardly influences the QCM signal, which remains stable.

Since we do not see changes in the signal upon flushing with concentrated BSA, we can also conclude that, in the ***B***-***M***-***E*** coatings, all binding blocks ***B*** are properly oriented toward the silica surface, as designed. In fact, for the more weakly bound ***B***-***E*** diblocks, we observed strong displacement caused by BSA, indicating that the negatively charged BSA molecules compete effectively with the negatively charged silica. This also indicates a strong interaction between the ***B*** blocks and BSA. In the ***B***-***M***-***E*** coatings, any exposed binding blocks ***B*** would have also led to strong interactions with BSA, which we did not observe.

## Concluding Remarks

Our work shows that it is possible to design, produce, and purify self-assembling polypeptide brushes that harness multivalent binding of SBPs for attachment to solid surfaces. The number of successful designs is strongly limited by practical constraints such as polypeptide toxicity, low expression levels, and low solubility. However, we also showed that the design with sequence ***B*****^*RT*^**-***E^S^***_3_-***M^HR00C3_2^***-***E^S^***_40_ has very promising properties, forming highly stable and antifouling polypeptide brushes on silica surfaces.

Given the initial success of our ***B***-***M***-***E*** design as an antifoulant against concentrated solutions of BSA in PBS, the following step would be to evaluate complex biological fluids such as diluted serum. In fact, the antifouling behavior against a single prominent serum protein such as BSA does not guarantee similar results with a complex mixture of biomolecules.^38,39^ Hence, further changes to our original design may still be necessary. These could include changing the length or nature of the ***E*** block.

While PLL-*g*-PEG brushes are already adequate for many applications, we believe that recombinant antifouling polypeptides may offer advantages for specific cases. In particular, the ***B***-***M***-***E*** triblock sequence can be directly used as a combined adhesion and antifouling tag for displaying proteins on surfaces. Also, given the wealth of data on SBPs suitable for other surfaces, we expect it will be relatively straightforward to develop ***B***-***M***-***E*** triblocks that strongly adhere to, for example, metals and plastics.

## Supporting Information Available

DNA and peptide sequences and SDS-PAGE analysis of expression and purification of all constructs; QCM-D results on the salt stability of brushes formed by ***B***_*n*_-***E*** diblock polypeptides with tandem-repeated SBPs for *n* = 1 and 2; and SEC of ***B***-***M***-***E*** triblocks at high salt (PDF)
