# Supplementary material for: Design of Polypeptides Self-Assembling into Antifouling Coatings: Exploiting Multivalency
Source: Biomacromolecules. 2022 Aug 11;23(9):3507–16. doi: 10.1021/acs.biomac.2c00170 (PMC9472226; doi:10.1021/acs.biomac.2c00170)
Supplement: Supplementary file 1 — bm2c00170_si_001.pdf [file bm2c00170_si_001.pdf]

Supporting Information

Design of polypeptides self-assembling into antifouling coatings: exploiting multivalency

*Nicolò Alvisi[1]; Chuanbao Zheng[1]; Meike Lokker[1]; Victor Boekestein[1]; Rob de Haas[1]; Bauke Albada[2]; Renko de Vries[1]**

AUTHOR ADDRESS. [1]Department of Physical Chemistry and Soft Matter, Wageningen University & Research, Stippeneng 4, 6708 WE, Wageningen, The Netherlands. [2]Laboratory of Organic Chemistry, Wageningen University & Research, Stippeneng 4, 6708 WE, Wageningen, The Netherlands.

Contents

Table S1. DNA sequences of all constructs.

Table S2. Amino acid sequences of all constructs.

Figure S1. Expression and purification of polypeptides $\boldsymbol{B_n}$-***E*** with tandem repeated SBPs.

Figure S2. Stability of brushes formed on silica surfaces by $\boldsymbol{B_n}$-***E*** polymers with $n = 1$ and 2.

Figure S3. Expression and purification of triblock constructs ***B-M-E***.

Figure S4. Expression and purification of ***B-M*** di-block construct

Figure S5. Size exclusion chromatography of triblock constructs ***B-M-E*** at high salt

Table S1. DNA sequences of all constructs

Construct name	DNA sequence
B^{RT}-E^{S}_{20}	AGTTGTCAGTTTGCTGGTTCAGGTAAAGGTTCCTCACGCTAGGA<br>TCCAAATAATTTTGTTTAACTTTAAGAAGGAGGAGTACATATGG<br>GTTCGAGCCATCATCACCATCACCATTCTTCGGGTGGTTCGGGT<br>GTTCCGGGGTCTGGCGTACCGGGTTCAGGAGTTCCACGCACGCA<br>CCGTAAGCGCACCCACCGTAAACGTACACATCGTAAACGTACGC<br>ACCGCAAAGGATGATAATAATGATCTTCAGGATCACTCGAGCTG<br>CTCACGGAACTTACTGTATGAGTAGTGATTTGAAAG
B^{RT}-E^{S}_{3} -<br>B^{RT}-E^{S}_{20}	GGATCCAAATAATTTTGTTTAACTTTAAGAAGGAGGAGTACATA<br>TGGGTTCGAGCCATCATCACCATCACCATTCTTCGGGTGGTTCG<br>GGTGTTCCGGGGTCTGGCGTACCGGGTTCAGGAGTTCCACGCAC<br>GCACCGTAAGCGCACCCACCGTAAACGTACACATCGTAAACGTA<br>CGCACCGCAAAGGCTCCGGTGTCCCTGGTTCTGGCGTGCCAGGT<br>AGCGGGGTACCGCGCACACACCGGAAGCGTACGCATCGGAAAC<br>GGACCCATCGTAAACGGACGCATCGTAAAGGATGATAATAATG<br>ATCTTCAGGATCACTCGAGC
B^{RT}-E^{S}_{3} -<br>B^{RT}-E^{S}_{3} -<br>B^{RT}-E^{S}_{20}	GGATCCAAATAATTTTGTTTAACTTTAAGAAGGAGGAGTACATA<br>TGGGTTCGAGCCATCATCACCATCACCATTCTTCGGGTGGTTCG<br>GGTGTTCCGGGGTCTGGCGTACCGGGTTCAGGAGTTCCACGCAC<br>GCACCGTAAGCGCACCCACCGTAAACGTACACATCGTAAACGTA<br>CGCACCGCAAAGGCTCCGGTGTCCCTGGTTCTGGCGTGCCAGGT<br>AGCGGGGTACCGCGCACACACCGGAAGCGTACGCATCGGAAAC<br>GGACCCATCGTAAACGGACGCATCGTAAAGGAAGCGGTGTTCC<br>AGGGAGTGGCGTTCCAGGCAGTGGTGTGCCGCGGACTCATCGCA<br>AACGTACCCACCGGAAACGCACGCATCGGAAACGTACCCACCG<br>CAAGGGATGATAATAATGATCTTCAGGATCACTCGAGC
E^{S}_{20}	GGATCCAAATAATTTTGTTTAACTTTAAGAAGGAGGAGTACATA<br>TGGGAAGCGGCGTCCCAGGCTCGGGCGTACCTGGATCTGGCGTT<br>CCAGGCAGCGGTGTTCCAGGGAGTGGCGTTCCGGGTTCAGGCGT<br>TCCTGGCTCAGGTGTGCCGGGTTCCGGTGTGCCGGGCAGTGGAG

	TACCAGGTAGCGGGGTTCCTGGAAGTGGTGTACCAGGTTCTGGC<br>GTCCCAGGGAGCGGCGTCCCGGGTTCTGGTGTGCCAGGAAGTGG<br>GGTTCCGGGGTCAGGTGTCCCAGGCAGTGGTGTTCCGGGGTCCG<br>GTGTACCGGGCAGTGGGGTACCTGGGTCGGGAGTGCCGGGCTG<br>CTGATAATGATCTTCAGGATCACTCGAG
foldon	AATAATTTTGTTTAACTTTAAGAAGGAGGAGTACATATGCGTAC<br>CCATCGGAAACGCACTCACCGCAAACGCACACACCGGAAGCGT<br>ACGCATCGCAAGGGCTATATTCCGGAAGCGCCGCGCGATGGCC<br>AGGCGTATGTGCGCAAAGATGGCGAATGGGTGCTGCTGAGCAC<br>CTTTCTGTCGGGAGTGCCTGGATCGGGCGTTCCTGGGTCCGGAG<br>TACCTGGCTCGGGAGTACCGGGTTCTGGCGTTCCAGGTAGTGGC<br>GTCCCAGGGAGTGGGGTACCTGGGTCGGGTGTACCGGGCTCTGG<br>TGTGCCTGGTTCGGGAGTCCCAGGTTCCGGAGTGCCAGGATCAG<br>GCGTACCAGGCTCAGGTGTCCCTGGTTCAGGAGTGCCGGGAAGT<br>GGGGTCCCTGGATCTGGTGTCCCAGGATCTGGAGTTCCGGGATC<br>AGGTGTGCCAGGTTCTGGAGTGCCCGGATCTGGGGTGCCTGGGA<br>GCGGCGTACCTGGTAGTGGGGTTCCAGGCTCCGGTGTACCCGGA<br>TCAGGGGTACCAGGTTCAGGTGTACCTGGATCAGGAGTCCCGGG<br>TAGTGGAGTACCAGGGTCAGGTGTTCCAGGGTCTGGAGTCCCTG<br>GCAGTGGTGTACCAGGATCCGGAGTCCCCGGAAGCGGTGTTCCC<br>GGTAGCGGAGTTCCAGGATCGGGGGTACCGGGGTCAGGAGTTC<br>CTGGATCCGGCGTGCCAGGCAGCGGGGTACCCGGCTCAGGAGT<br>ACCCGGTTCAGGGGTTCCTGGTTCTGGTGTTCCTGGCTGTTGATA<br>ATAATGATCTTCAGGATCC
I53.50A	TCTAGAAATAATTTTGTTTAACTTTAAGAAGGAGGAGTACATAT<br>GGGCCATCACCATCACCATCACGGGGGCTCGCGTACCCATCGGA<br>AACGCACTCACCGCAAACGCACACACCGGAAGCGTACGCATCG<br>CAAGATGAAAATGGAAGAACTGTTTAAAAAACATAAAATTGTG<br>GCGGTGCTGCGCGCGAACAGCGTGGAAGAAGCGATTGAAAAAG<br>CGGTGGCGGTGTTTGCGGGCGGCGTGCATCTGATTGAAATTACC<br>TTTACCGTGCCGGATGCGGATACCGTGATTAAAGCGCTGAGCGT

	GCTGAAAGAAAAAGGCGCGATTATTGGCGCGGGCACCGTGACC AGCGTGGAACAGGCGCGCAAAGCGGTGGAAAGCGGCGCGGAAT TTATTGTGAGCCCGCATCTGGATGAAGAAATTAGCCAGTTTGCG AAAGAAAAAGGCGTGTTTTATATGCCGGGCGTGATGACCCCGAC CGAACTGGTGAAAGCGATGAAACTGGGCCATGATATTCTGCTGC TGTTTCCGGGCGAAGTGGTGGGCCCGCAGTTTGTGAAAGCGATG AAAGGCCCGTTTCCGAACGTGAAATTTGTGCCGACCGGCGGCGT GAACCTGGATAACGTGGCGAAATGGTTTAAAGCGGGCGTGCTG GCGGTGGGCGTGGGCAAAGCGCTGGTGAAAGGCAAACCGGATG AAGTGCGCGAAAAAGCGAAAAAATTTGTGAAAAAAATTCGCGG CGCGACCGAAGGCAGCCTGGAAGGCTGATAATAATGATCTTCA GGATCCGAATTC
LSM-α	AATAATTTTGTTTAACTTTAAGAAGGAGGAGTACATATGCGTAC CCATCGGAAACGCACTCACCGCAAACGCACACACCGGAAGCGT ACGCATCGCAAGAGCCAGCGCGTGAACGTGCAGCGCCCGCTGG ATGCGCTGGGCAACAGCCTGAACAGCCCGGTGATTATTAAACTG AAAGGCGATCGCGAATTTCGCGGCGTGCTGAAAAGCTTTGATCT GCATATGAACCTGGTGCTGAACGATGCGGAAGAACTGGAAGAT GGCGAAGTGACCCGCCGCCTGGGCACCGTGCTGATTCGCGGCGA TAACATTGTGTATATTAGCCCGTCGGGAGTGCCTGGATCGGGCG TTCCTGGGTCCGGAGTACCTGGCTCGGGAGTACCGGGTTCTGGC GTTCCAGGTAGTGGCGTCCCAGGGAGTGGGGTACCTGGGTCGGG TGTACCGGGCTCTGGTGTGCCTGGTTCGGGAGTCCCAGGTTCCG GAGTGCCAGGATCAGGCGTACCAGGCTCAGGTGTCCCTGGTTCA GGAGTGCCGGGAAGTGGGGTCCCTGGATCTGGTGTCCCAGGATC TGGAGTTCCGGGATCAGGTGTGCCAGGTTCTGGAGTGCCCGGAT CTGGGGTGCCTGGGAGCGGCGTACCTGGTAGTGGGGTTCCAGGC TCCGGTGTACCCGGATCAGGGGTACCAGGTTCAGGTGTACCTGG ATCAGGAGTCCCGGGTAGTGGAGTACCAGGGTCAGGTGTTCCAG GGTCTGGAGTCCCTGGCAGTGGTGTACCAGGATCCGGAGTCCCC GGAAGCGGTGTTCCCGGTAGCGGAGTTCCAGGATCGGGGGTAC

	CGGGGTCAGGAGTTCCTGGATCCGGCGTGCCAGGCAGCGGGGT<br>ACCCGGCTCAGGAGTACCCGGTTCAGGGGTTCCTGGTTCTGGTG<br>TTCCTGGCTGTTGATAATAATGATCTTCAGGATCC
TRAP	AATAATTTTGTTTAACTTTAAGAAGGAGGAGTACATATGTATAC<br>CAACAGCGATTTTGTGGTGATTAAAGCGCTGGAAGATGGCGTGA<br>ACGTGATTGGCCTGACCCGCGGCGCGGATACCCGCTTTCATCAT<br>AGCGAAAAACTGGATAAAGGCGAAGTGCTGATTGCGCAGTTTA<br>CCGAACATACCAGCGCGATTAAAGTGCGCGGCAAAGCGTATATT<br>CAGACCCGCCATGGCGTGATTGAAAGCGAAGGCAAAAAATCGG<br>GAGTGCCTGGATCGGGCGTTCCTGGGTCCGGAGTACCTGGCTCG<br>GGAGTACCGGGTTCTGGCGTTCCAGGTCGTACCCATCGGAAACG<br>CACTCACCGCAAACGCACACACCGGAAGCGTACGCATCGCAAG<br>TCGGGAGTGCCTGGATCGGGCGTTCCTGGGTCCGGAGTACCTGG<br>CTCGGGAGTACCGGGTTCTGGCGTTCCAGGTAGTGGCGTCCCAG<br>GGAGTGGGGTACCTGGGTCGGGTGTACCGGGCTCTGGTGTGCCT<br>GGTTCGGGAGTCCCAGGTTCCGGAGTGCCAGGATCAGGCGTACC<br>AGGCTCAGGTGTCCCTGGTTCAGGAGTGCCGGGAAGTGGGGTCC<br>CTGGATCTGGTGTCCCAGGATCTGGAGTTCCGGGATCAGGTGTG<br>CCAGGTTCTGGAGTGCCCGGATCTGGGGTGCCTGGGAGCGGCGT<br>ACCTGGTAGTGGGGTTCCAGGCTCCGGTGTACCCGGATCAGGGG<br>TACCAGGTTCAGGTGTACCTGGATCAGGAGTCCCGGGTAGTGGA<br>GTACCAGGGTCAGGTGTTCCAGGGTCTGGAGTCCCTGGCAGTGG<br>TGTACCAGGATCCGGAGTCCCCGGAAGCGGTGTTCCCGGTAGCG<br>GAGTTCCAGGATCGGGGGTACCGGGGTCAGGAGTTCCTGGATCC<br>GGCGTGCCAGGCAGCGGGGTACCCGGCTCAGGAGTACCCGGTT<br>CAGGGGTTCCTGGTTCTGGTGTTCCTGGCTGTTGATAATAATGAT<br>CTTCAGGATCC
1na0C3_3	AATAATTTTGTTTAACTTTAAGAAGGAGGAGTACATATGGGTTC<br>CAGCCACCACCATCATCACCATTCGAGCGGAATGAATCTCGCGG<br>AGAAGATGTATAAGGCGGGCAACGCAATGTATCGTAAAGGGCA<br>GTACACCATCGCGATTATTGCGTATACTCTGGCACTGCTGAAAG

	ACCCTAATAACGCAGAAGCGTGGTATAACTTAGGCAATGCCGCC TACAAGAAGGGTGAGTACGATGAGGCAATTGAAGCATATCAGA AAGCGCTGGAATTAGATCCGAATAACGCGGAAGCCTGGTATAA TCTCGGTAATGCGTATTATAAGCAGGGCGATTATGACGAAGCTA TCGAATATTACCAGAAAGCCTTGGAGTTAGATCCAAATAATGCC GAAGCTAAACAGAATTTGGGGAACGCCAAACAGAAACAGGGCC TGGGATCAGGCGTGCCGGGCTCTGGTGTTCCTGGTAGTGGTGTC CCACGTACCCATCGCAAACGCACCCATCGGAAACGGACGCATC GTAAGCGTACACACCGCAAGGGCAGCGGTGTTCCGGGCTCTGGT GTCCCAGGTTCCGGCGTACCGGGCAGCGGTGTTCCTGGTTCTGG CGTGCCGGGCAGCGGTGTTCCGGGCTCTGGTGTCCCAGGTTCCG GCGTACCGGGCAGCGGTGTTCCTGGTTCTGGCGTGCCGGGCAGC GGTGTTCCGGGCTCTGGTGTCCCAGGTTCCGGCGTACCGGGCAG CGGTGTTCCTGGTTCTGGCGTGCCGGGCAGCGGTGTTCCGGGCT CTGGTGTCCCAGGTTCCGGCGTACCGGGCAGCGGTGTTCCTGGT TCTGGCGTGCCGGGCAGCGGTGTTCCGGGCTCTGGTGTCCCAGG TTCCGGCGTACCGGGCAGCGGTGTTCCTGGTTCTGGCGTGCCGG GCAGCGGTGTTCCGGGCTCTGGTGTCCCAGGTTCCGGCGTACCG GGCAGCGGTGTTCCTGGTTCTGGCGTGCCGGGCAGCGGTGTTCC GGGCTCTGGTGTCCCAGGTTCCGGCGTACCGGGCAGCGGTGTTC CTGGTTCTGGCGTGCCGGGCAGCGGTGTTCCGGGCTCTGGTGTC CCAGGTTCCGGCGTACCGGGCAGCGGTGTTCCTGGTTCTGGCGT GCCGGGCTGATAATAATGATCTTCAGGATCC
HR00C3_2	AATAATTTTGTTTAACTTTAAGAAGGAGGAGTACATATGGGATC GAGCCATCACCATCACCATCATTCGAGCGGACGGACGCACCGTA AACGCACCCACCGGAAACGTACCCATCGGAAACGCACTCATCG CAAAGGCTCTGGCGTCCCAGGTTCCGGTGTACCTGGATCTGGCG TACCGATGATCGAGGAAGTCGTAGCTGAGATGATCGACATCCTG GCGGAGTCCAGCAAGAAAAGCATCGAGGAATTAGCGCGTGCTG CCGATAACAAGACGACCGAGAAGGCCGTGGCCGAAGCGATTGA GGAAATTGCCCGCCTGGCGACCGCAGCGATTCAGCTGATCGAGG

	CATTAGCCAAGAATTTGGCGTCTGAGGAATTTATGGCCCGTGCA<br>ATTTCCGCCATCGCTGAATTGGCAAAGAAGGCAATTGAAGCAAT<br>CTATCGCTTGGCTGACAATCACACCACGGACACCTTCATGGCAC<br>GCGCCATCGCGGCGATCGCGAACCTCGCCGTTACTGCAATCTTG<br>GCTATCGCCGCATTAGCATCGAATCATACAACCGAGGAATTCAT<br>GGCGCGTGCGATTTCCGCTATTGCAGAACTCGCGAAGAAAGCCA<br>TCGAAGCCATTTATCGCCTGGCAGATAACCATACCACGGATAAA<br>TTTATGGCGGCTGCTATCGAGGCCATCGCACTGCTTGCCACGCT<br>GGCGATTCTGGCCATTGCCCTGCTTGCGAGTAATCACACAACTG<br>AGGAATTCATGGCCAAAGCCATTAGTGCGATTGCGGAGTTGGCG<br>AAGAAGGCGATCGAAGCTATTTACCGTTTGGCGGATAACCACAC<br>AAGTCCAACCTATATCGAGAAGGCTATTGAGGCAATTGAGAAG<br>ATTGCGCGCAAAGCGATTAAGGCCATTGAAATGCTGGCCAAGA<br>ACATCACCACGGAAGAATACAAAGAGAAAGCAAAATCGGCGAT<br>TGATGAAATCCGGGAAAAGGCGAAGGAAGCGATCAAACGTTTA<br>GAAGACAATCGTACCGGCAGCGGTGTTCCGGGCTCTGGTGTCCC<br>AGGTTCCGGCGTACCGGGCAGCGGTGTTCCTGGTTCTGGCGTGC<br>CGGGCAGCGGTGTTCCGGGCTCTGGTGTCCCAGGTTCCGGCGTA<br>CCGGGCAGCGGTGTTCCTGGTTCTGGCGTGCCGGGCAGCGGTGT<br>TCCGGGCTCTGGTGTCCCAGGTTCCGGCGTACCGGGCAGCGGTG<br>TTCCTGGTTCTGGCGTGCCGGGCAGCGGTGTTCCGGGCTCTGGT<br>GTCCCAGGTTCCGGCGTACCGGGCAGCGGTGTTCCTGGTTCTGG<br>CGTGCCGGGCAGCGGTGTTCCGGGCTCTGGTGTCCCAGGTTCCG<br>GCGTACCGGGCAGCGGTGTTCCTGGTTCTGGCGTGCCGGGCAGC<br>GGTGTTCCGGGCTCTGGTGTCCCAGGTTCCGGCGTACCGGGCAG<br>CGGTGTTCCTGGTTCTGGCGTGCCGGGCAGCGGTGTTCCGGGCT<br>CTGGTGTCCCAGGTTCCGGCGTACCGGGCAGCGGTGTTCCTGGT<br>TCTGGCGTGCCGGGCAGCGGTGTTCCGGGCTCTGGTGTCCCAGG<br>TTCCGGCGTACCGGGCAGCGGTGTTCCTGGTTCTGGCGTGCCGG<br>GCTGATAATAATGATCTTCAGGATCC

ank1C4_2	AATAATTTTGTTTAACTTTAAGAAGGAGGAGTACATATGGGGAG<br>TTCTCACCACCATCATCATCATAGCAGCGGCATGTCAGAGGACG<br>GCGAACTGCTGATCTTAGCCGCCGAGCTCGGCATCGCAGAGGCG<br>GTGCGTATGCTGATTGAACAGGGTGCGGACGTAAACGCATCCGA<br>TGACGATGGACGGACCCCGTTGCACCACGCGGCCGAGAACGGG<br>CACTTGGCAGTGGTTCTGCTTCTGTTATTGAAAGGCGCAGACGT<br>GAACGCAAAGGACTCCGATGGCCGCACCCCTCTCCACCACGCGG<br>CAGAAAACGGCCACAAAACCGTCGTGCTTTTACTGATTCTCATG<br>GGCGCAGACGTGAATGCGAAGGACTCCGACGGCCGCACCCCAC<br>TGCATCACGCGGCGGAAAACGGTCATAAGGAAGTAGTGAAACT<br>GCTCATTCGTAAAGGCGCCGACGTGAATACGAGTGATTCTGACG<br>GACGCACCCCGCTCGATTTGGCTCGCGAACACGGCAACGAGGA<br>AGTTGTTAAATTACTGGAGAAACAACTGGGTTCAGGGGTACCAG<br>GGTCTGGCGTGCCGGGTAGCGGGGTTCCGCGTACCCATCGCAAG<br>CGCACGCACCGCAAACGGACGCATCGCAAACGGACACATCGCA<br>AGGGCAGCGGTGTTCCGGGCTCTGGTGTCCCAGGTTCCGGCGTA<br>CCGGGCAGCGGTGTTCCTGGTTCTGGCGTGCCGGGCAGCGGTGT<br>TCCGGGCTCTGGTGTCCCAGGTTCCGGCGTACCGGGCAGCGGTG<br>TTCCTGGTTCTGGCGTGCCGGGCAGCGGTGTTCCGGGCTCTGGT<br>GTCCCAGGTTCCGGCGTACCGGGCAGCGGTGTTCCTGGTTCTGG<br>CGTGCCGGGCAGCGGTGTTCCGGGCTCTGGTGTCCCAGGTTCCG<br>GCGTACCGGGCAGCGGTGTTCCTGGTTCTGGCGTGCCGGGCAGC<br>GGTGTTCCGGGCTCTGGTGTCCCAGGTTCCGGCGTACCGGGCAG<br>CGGTGTTCCTGGTTCTGGCGTGCCGGGCAGCGGTGTTCCGGGCT<br>CTGGTGTCCCAGGTTCCGGCGTACCGGGCAGCGGTGTTCCTGGT<br>TCTGGCGTGCCGGGCAGCGGTGTTCCGGGCTCTGGTGTCCCAGG<br>TTCCGGCGTACCGGGCAGCGGTGTTCCTGGTTCTGGCGTGCCGG<br>GCAGCGGTGTTCCGGGCTCTGGTGTCCCAGGTTCCGGCGTACCG<br>GGCAGCGGTGTTCCTGGTTCTGGCGTGCCGGGCTGATAATAATG<br>ATCTTCAGGATCC

E^{S}_{40}	GAGGAGATACATATGGTCGGGAGTGCCTGGATCGGGCGTTCCTG<br>GGTCCGGAGTACCTGGCTCGGGAGTACCGGGTTCTGGCGTTCCA<br>GGTAGTGGCGTCCCAGGGAGTGGGGTACCTGGGTCGGGTGTACC<br>GGGCTCTGGTGTGCCTGGTTCGGGAGTCCCAGGTTCCGGAGTGC<br>CAGGATCAGGCGTACCAGGCTCAGGTGTCCCTGGTTCAGGAGTG<br>CCGGGAAGTGGGGTCCCTGGATCTGGTGTCCCAGGATCTGGAGT<br>TCCGGGATCAGGTGTGCCAGGTTCTGGAGTGCCCGGATCTGGGG<br>TGCCTGGGAGCGGCGTACCTGGTAGTGGGGTTCCAGGCTCCGGT<br>GTACCCGGATCAGGGGTACCAGGTTCAGGTGTACCTGGATCAGG<br>AGTCCCGGGTAGTGGAGTACCAGGGTCAGGTGTTCCAGGGTCTG<br>GAGTCCCTGGCAGTGGTGTACCAGGATCCGGAGTCCCCGGAAGC<br>GGTGTTCCCGGTAGCGGAGTTCCAGGATCGGGGGTACCGGGGTC<br>AGGAGTTCCTGGATCCGGCGTGCCAGGCAGCGGGGTACCCGGCT<br>CAGGAGTACCCGGTTCAGGGGTTCCTGGTTCTGGTGTTCCTGGC<br>TGATAATAATGATCTTCAGGAATTC

Table S2. Amino acid sequences of all constructs

Construct	Amino acid sequence
B^{RT}-E^{S}_{40}	MGSSHHHHHHSSGGSGVPGSGVPGSGVPRTHRKRTHRKRTHRKRT<br>HRKGSGVPGSGVPGSGVPGSGVPGSGVPGSGVPGSGVPGSGVPGS<br>GVPGSGVPGSGVPGSGVPGSGVPGSGVPGSGVPGSGVPGSGVPGSG<br>VPGSGVPGSGVPGSGVPGSGVPGSGVPGSGVPGSGVPGSGVPGSGV<br>PGSGVPGSGVPGSGVPGSGVPGSGVPGSGVPGSGVPGSGVPGSGVP<br>GSGVPGSGVPGSGVPGSGVPGC
B^{RT}-E^{S}_{3}-B^{RT}-E^{S}_{40}	MGSSHHHHHHSSGGSGVPGSGVPGSGVPRTHRKRTHRKRTHRKRT<br>HRKGSGVPGSGVPGSGVPRTHRKRTHRKRTHRKRTHRKGSGVPGS<br>GVPGSGVPGSGVPGSGVPGSGVPGSGVPGSGVPGSGVPGSGVPGSG<br>VPGSGVPGSGVPGSGVPGSGVPGSGVPGSGVPGSGVPGSGVPGSGV

	PGSGVPGSGVPGSGVPGSGVPGSGVPGSGVPGSGVPGSGVPGSGVP<br>GSGVPGSGVPGSGVPGSGVPGSGVPGSGVPGSGVPGSGVPGSGVPG<br>SGVPGSGVPGWPC
$\boldsymbol{B}^{RT}$-$\boldsymbol{E}^{S}_{3}$-$\boldsymbol{B}^{RT}$-$\boldsymbol{E}^{S}_{3}$-$\boldsymbol{B}^{RT}$-$\boldsymbol{E}^{S}_{40}$	MGSSHHHHHHSSGGSGVPGSGVPGSGVPRTHRKRTHRKRTHRKRT<br>HRKGSGVPGSGVPGSGVPRTHRKRTHRKRTHRKRTHRKGSGVPGS<br>GVPGSGVPRTHRKRTHRKRTHRKRTHRKGSGVPGSGVPGSGVPGS<br>GVPGSGVPGSGVPGSGVPGSGVPGSGVPGSGVPGSGVPGSGVPGSG<br>VPGSGVPGSGVPGSGVPGSGVPGSGVPGSGVPGSGVPGSGVPGSGV<br>PGSGVPGSGVPGSGVPGSGVPGSGVPGSGVPGSGVPGSGVPGSGVP<br>GSGVPGSGVPGSGVPGSGVPGSGVPGSGVPGSGVPGSGVPGSGVPG<br>C
$\boldsymbol{B}^{RT}$- $\boldsymbol{M}^{foldon}$-$\boldsymbol{E}^{S}_{40}$	MRTHRKRTHRKRTHRKRTHRKGYIPEAPRDGQAYVRKDGEWVLL<br>STFLSGVPGSGVPGSGVPGSGVPGSGVPGSGVPGSGVPGSGVPGSG<br>VPGSGVPGSGVPGSGVPGSGVPGSGVPGSGVPGSGVPGSGVPGSGV<br>PGSGVPGSGVPGSGVPGSGVPGSGVPGSGVPGSGVPGSGVPGSGVP<br>GSGVPGSGVPGSGVPGSGVPGSGVPGSGVPGSGVPGSGVPGSGVPG<br>SGVPGSGVPGSGVPGSGVPG
$\boldsymbol{B}^{RT}$- $\boldsymbol{E}^{S}_{5}$-$\boldsymbol{M}^{I53.50A}$-$\boldsymbol{E}^{S}_{40}$	MGHHHHHHGGSRTHRKRTHRKRTHRKRTHRKSGVPGSGVPGSGV<br>PGSGVPGSGVPGMKMEELFKKHKIVAVLRANSVEEAIEKAVAVFA<br>GGVHLIEITFTVPDADTVIKALSVLKEKGAIIGAGTVTSVEQARKAV<br>ESGAEFIVSPHLDEEISQFAKEKGVFYMPGVMTPTELVKAMKLGHD<br>ILLLFPGEVVGPQFVKAMKGPFPNVKFVPTGGVNLDNVAKWFKAG<br>VLAVGVGKALVKGKPDEVREKAKKFVKKIRGATEGSLEGSGVPGS<br>GVPGSGVPGSGVPGSGVPGSGVPGSGVPGSGVPGSGVPGSGVPGSG<br>VPGSGVPGSGVPGSGVPGSGVPGSGVPGSGVPGSGVPGSGVPGSGV<br>PGSGVPGSGVPGSGVPGSGVPGSGVPGSGVPGSGVPGSGVPGSGVP<br>GSGVPGSGVPGSGVPGSGVPGSGVPGSGVPGSGVPGSGVPGSGVPG<br>SGVPGSGVPG

$\boldsymbol{B}^{RT}$- $\boldsymbol{E}^{S}{}_{3}$-<br>$\boldsymbol{M}^{LSM\text{-}\alpha}$-$\boldsymbol{E}^{S}{}_{40}$	MGHHHHHHGGSRTHRKRTHRKRTHRKRTHRKGSGVPGSGVPGSG<br>VPSQRVNVQRPLDALGNSLNSPVIIKLKGDREFRGVLKSFDLHMNL<br>VLNDAEELEDGEVTRRLGTVLIRGDNIVYISPSGVPGSGVPGSGVPG<br>SGVPGSGVPGSGVPGSGVPGSGVPGSGVPGSGVPGSGVPGSGVPGS<br>GVPGSGVPGSGVPGSGVPGSGVPGSGVPGSGVPGSGVPGSGVPGSG<br>VPGSGVPGSGVPGSGVPGSGVPGSGVPGSGVPGSGVPGSGVPGSGV<br>PGSGVPGSGVPGSGVPGSGVPGSGVPGSGVPGSGVPGSGVPGSGVP<br>G
$\boldsymbol{M}^{TRAP}$-$\boldsymbol{E}^{S}{}_{3}$-<br>$\boldsymbol{B}^{RT}$-$\boldsymbol{E}^{S}{}_{40}$	MGHHHHHHGGSYTNSDFVVIKALEDGVNVIGLTRGADTRFHHSEK<br>LDKGEVLIAQFTEHTSAIKVRGKAYIQTRHGVIESEGKKSGVPGSGV<br>PGSGVPGSGVPGSGVPGRTHRKRTHRKRTHRKRTHRKSGVPGSGV<br>PGSGVPGSGVPGSGVPGSGVPGSGVPGSGVPGSGVPGSGVPGSGVP<br>GSGVPGSGVPGSGVPGSGVPGSGVPGSGVPGSGVPGSGVPGSGVPG<br>SGVPGSGVPGSGVPGSGVPGSGVPGSGVPGSGVPGSGVPGSGVPGS<br>GVPGSGVPGSGVPGSGVPGSGVPGSGVPGSGVPGSGVPGSGVPGSG<br>VPGSGVPG
$\boldsymbol{M}^{1na0C3\_3}$-<br>$\boldsymbol{E}^{S}{}_{3}$-$\boldsymbol{B}^{RT}$-$\boldsymbol{E}^{S}{}_{40}$	MGSSHHHHHHSSGMNLAEKMYKAGNAMYRKGQYTIAIIAYTLAL<br>LKDPNNAEAWYNLGNAAYKKGEYDEAIEAYQKALELDPNNAEA<br>WYNLGNAYYKQGDYDEAIEYYQKALELDPNNAEAKQNLGNAKQ<br>KQGLGSGVPGSGVPGSGVPRTHRKRTHRKRTHRKRTHRKGSGVPG<br>SGVPGSGVPGSGVPGSGVPGSGVPGSGVPGSGVPGSGVPGSGVPGS<br>GVPGSGVPGSGVPGSGVPGSGVPGSGVPGSGVPGSGVPGSGVPGSG<br>VPGSGVPGSGVPGSGVPGSGVPGSGVPGSGVPGSGVPGSGVPGSGV<br>PGSGVPGSGVPGSGVPGSGVPGSGVPGSGVPGSGVPGSGVPGSGVP<br>GSGVPGSGVPG
$\boldsymbol{B}^{RT}$-$\boldsymbol{E}^{S}{}_{3}$-<br>$\boldsymbol{M}^{HR00C3\_2}$-<br>$\boldsymbol{E}^{S}{}_{40}$	MGSSHHHHHHSSGRTHRKRTHRKRTHRKRTHRKGSGVPGSGVPGS<br>GVPMIEEVVAEMIDILAESSKKSIEELARAADNKTTEKAVAEAIEEI<br>ARLATAAIQLIEALAKNLASEEFMARAISAIAELAKKAIEAIYRLAD<br>NHTTDTFMARAIAAIANLAVTAILAIAALASNHTTEEFMARAISAIA<br>ELAKKAIEAIYRLADNHTTDKFMAAAIEAIALLATLAILAIALLASN

	HTTEEFMAKAISAIAELAKKAIEAIYRLADNHTSPTYIEKAIEAIEKIA<br>RKAIKAIEMLAKNITTEEYKEKAKSAIDEIREKAKEAIKRLEDNRTG<br>SGVPGSGVPGSGVPGSGVPGSGVPGSGVPGSGVPGSGVPGSGVPGS<br>GVPGSGVPGSGVPGSGVPGSGVPGSGVPGSGVPGSGVPGSGVPGSG<br>VPGSGVPGSGVPGSGVPGSGVPGSGVPGSGVPGSGVPGSGVPGSGV<br>PGSGVPGSGVPGSGVPGSGVPGSGVPGSGVPGSGVPGSGVPGSGVP<br>GSGVPGSGVPGSGVPGSGVPGSGVPGSGVPG
$\boldsymbol{M}^{ank1C4\_2}$-$\boldsymbol{E}^{S}_{3}$-$\boldsymbol{B}^{RT}$-$\boldsymbol{E}^{S}_{40}$	MGSSHHHHHHSSGMSEDGELLILAAELGIAEAVRMLIEQGADVNA<br>SDDDGRTPLHHAAENGHLAVVLLLLLKGADVNAKDSDGRTPLHH<br>AAENGHKTVVLLLILMGADVNAKDSDGRTPLHHAAENGHKEVVK<br>LLIRKGADVNTSDSDGRTPLDLAREHGNEEVVKLLEKQLGSGVPGS<br>GVPGSGVPRTHRKRTHRKRTHRKRTHRKGSGVPGSGVPGSGVPGS<br>GVPGSGVPGSGVPGSGVPGSGVPGSGVPGSGVPGSGVPGSGVPGSG<br>VPGSGVPGSGVPGSGVPGSGVPGSGVPGSGVPGSGVPGSGVPGSGV<br>PGSGVPGSGVPGSGVPGSGVPGSGVPGSGVPGSGVPGSGVPGSGVP<br>GSGVPGSGVPGSGVPGSGVPGSGVPGSGVPGSGVPGSGVPGSGVPG

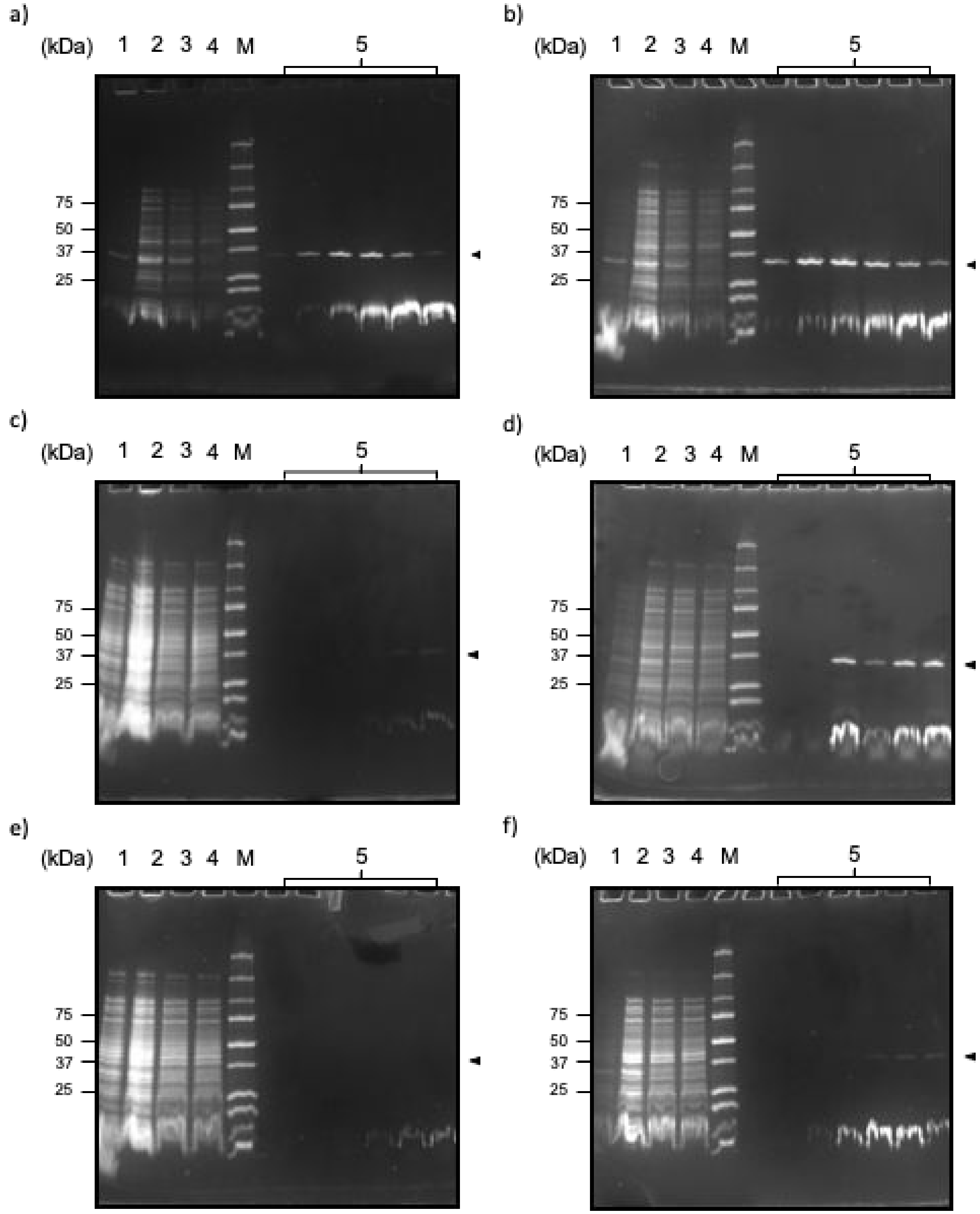

Figure S1. Expression and purification of polypeptides $\boldsymbol{B_n}$-$\boldsymbol{E}$ with tandem repeated SBPs: $\boldsymbol{B}^{RT}$-$\boldsymbol{E}^{S}{}_{40}$ (n = 1), $\boldsymbol{B}^{RT}$-$\boldsymbol{E}^{S}{}_{3}$ -$\boldsymbol{B}^{RT}$ -$\boldsymbol{E}^{S}{}_{40}$ (n = 2) and $\boldsymbol{B}^{RT}$-$\boldsymbol{E}^{S}{}_{3}$-$\boldsymbol{B}^{RT}$-$\boldsymbol{E}^{S}{}_{3}$-$\boldsymbol{B}^{RT}$-$\boldsymbol{E}^{S}{}_{40}$ (n = 3). SDS-PAGE

analysis. Lane 1: intact cells; lane 2: cell lysate; lane 3: insoluble lysate; lane 4: soluble lysate; lane 5: IMAC fractions; lane M: molecular marker. a) n = 1, LB medium, b) n = 1, LB medium + 0.5% D-glucose, c) n = 2, LB medium, d) n = 2, LB medium + 0.5% D-glucose, e) n = 3, LB medium, f) n = 3, LB medium + 0.5% D-glucose.

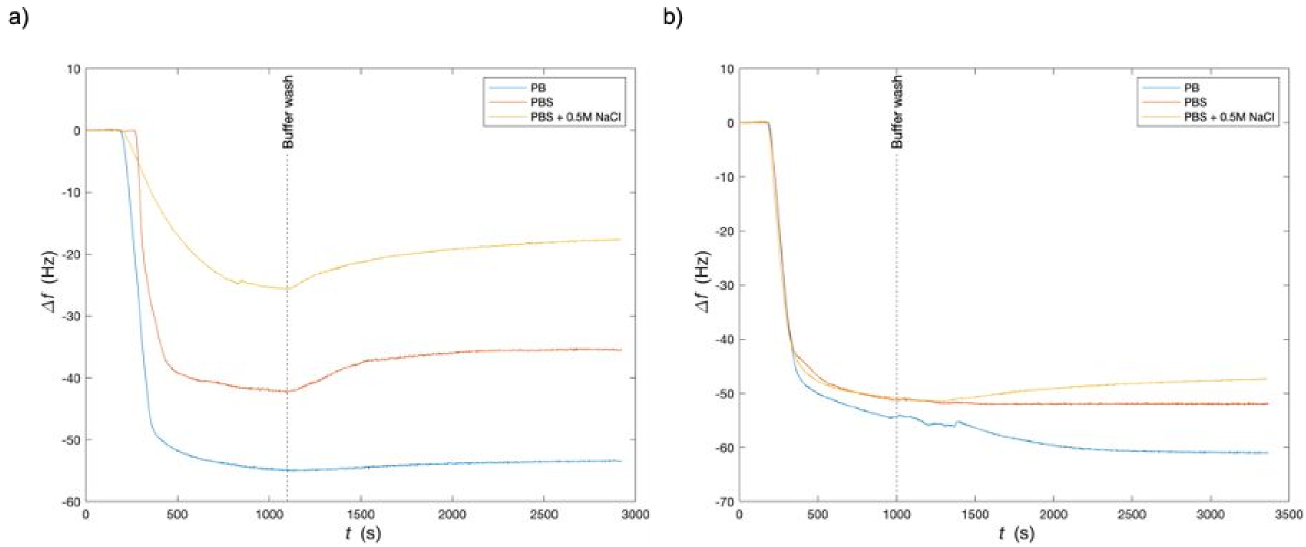

Figure S2. Stability of brushes formed on silica surfaces by $\boldsymbol{B}_n$-$\boldsymbol{E}$ polymers with n = 1 and 2 ($\boldsymbol{B}^{RT}$-$\boldsymbol{E}^{S}_{40}$ and $\boldsymbol{B}^{RT}$-$\boldsymbol{E}^{S}_{3}$-$\boldsymbol{B}^{RT}$-$\boldsymbol{E}^{S}_{40}$). Frequency change Δf (Hz) as measured by QCM, as a function of the time t(s) after the start of incubation of silica-coated quartz sensors with the polypeptides at a concentration of 5 µM Buffers used for brush formation and consecutive rinsing are the following: phosphate buffer (PB, blue), phosphate buffer saline (PBS, 150 mM NaCl, orange), and PBS + 0.5 M NaCl (yellow). Rinsing with buffer starts at the change in QCM signal following the vertical dashed line. a) $\boldsymbol{B}^{RT}$-$\boldsymbol{E}^{S}_{40}$; b) $\boldsymbol{B}^{RT}$-$\boldsymbol{E}^{S}_{3}$-$\boldsymbol{B}^{RT}$-$\boldsymbol{E}^{S}_{40}$.

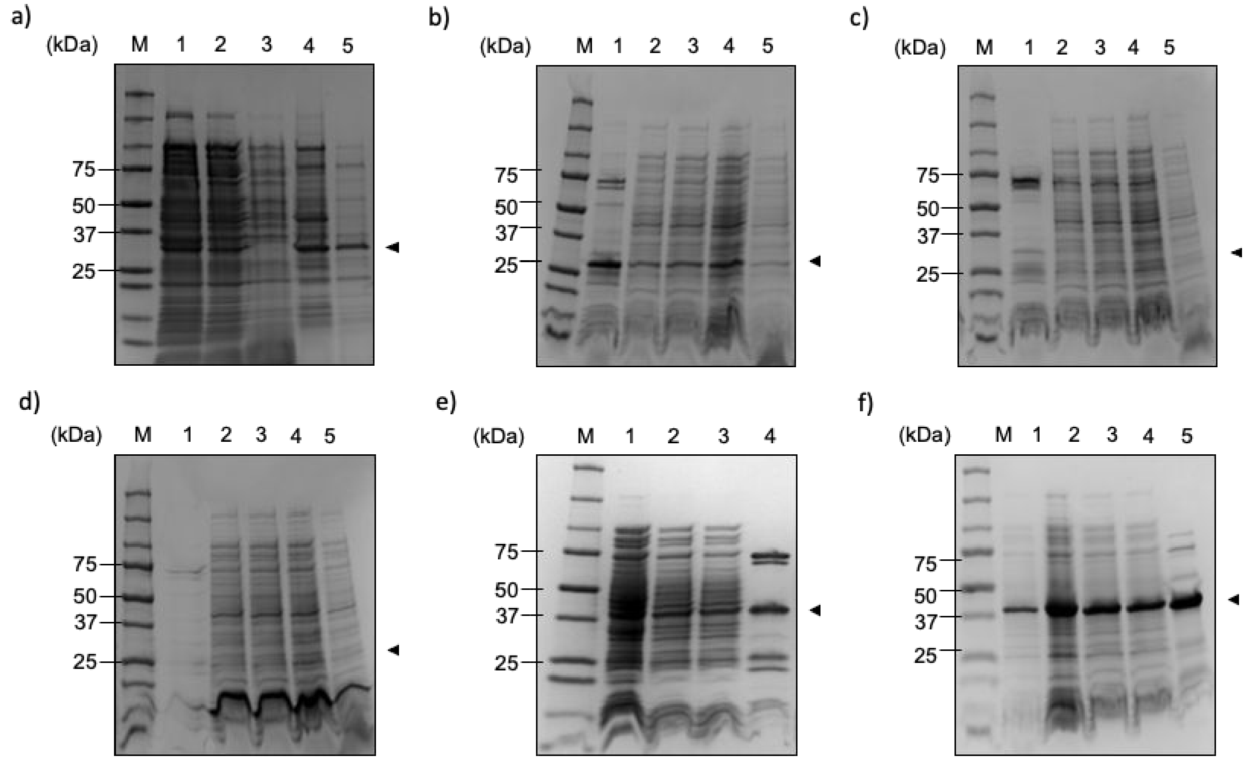

Figure S3. Expression and purification of triblock constructs ***B-M-E***. a) SDS-PAGE analysis of $\boldsymbol{B}^{RT}$- $\boldsymbol{M}^{foldon}$-$\boldsymbol{E}^{S}_{40}$. Lane 1: intact cells; lane 2: cell lysate; lane 3: insoluble lysate; lane 4: soluble lysate; lane 5: IMAC fractions; lane M: molecular marker. b) SDS-PAGE analysis of $\boldsymbol{B}^{RT}$- $\boldsymbol{E}^{S}_{5}$-$\boldsymbol{M}^{I53.50A}$-$\boldsymbol{E}^{S}_{40}$. Lane 1: IMAC fractions; lane 2: IMAC flow-through; lane 3: soluble lysate; lane 4: cell lysate; lane 5: intact cells; lane M: molecular marker. c) SDS-PAGE analysis of $\boldsymbol{B}^{RT}$- $\boldsymbol{E}^{S}_{3}$-$\boldsymbol{M}^{LSM\text{-}\alpha}$-$\boldsymbol{E}^{S}_{40}$. Lane 1: IMAC fractions; lane 2: IMAC flow-through; lane 3: soluble lysate; lane 4: cell lysate; lane 5: intact cells; lane M: molecular marker. d) SDS-PAGE analysis of $\boldsymbol{M}^{TRAP}$-$\boldsymbol{E}^{S}_{3}$-$\boldsymbol{B}^{RT}$-$\boldsymbol{E}^{S}_{40}$. Lane 1: intact cells; lane 2: cell lysate; lane 3: soluble lysate; lane 4: IMAC flow-through; lane 5: IMAC fractions; lane M: molecular marker. e) SDS-PAGE analysis of $\boldsymbol{M}^{1na0C3\_3}$-$\boldsymbol{E}^{S}_{3}$-$\boldsymbol{B}^{RT}$-

E^{S}_{40}. Lane 1: intact cells; lane 2: cell lysate; lane 3: soluble lysate; lane 4: IMAC fractions; lane M: molecular marker. f) SDS-PAGE analysis of M^{ank1C4_2}-E^{S}_{3}-B^{RT}-E^{S}_{40}. Lane 1: intact cells; lane 2: cell lysate; lane 3: soluble lysate; lane 4: IMAC flow-through; lane 5: IMAC fractions; lane M: molecular marker.

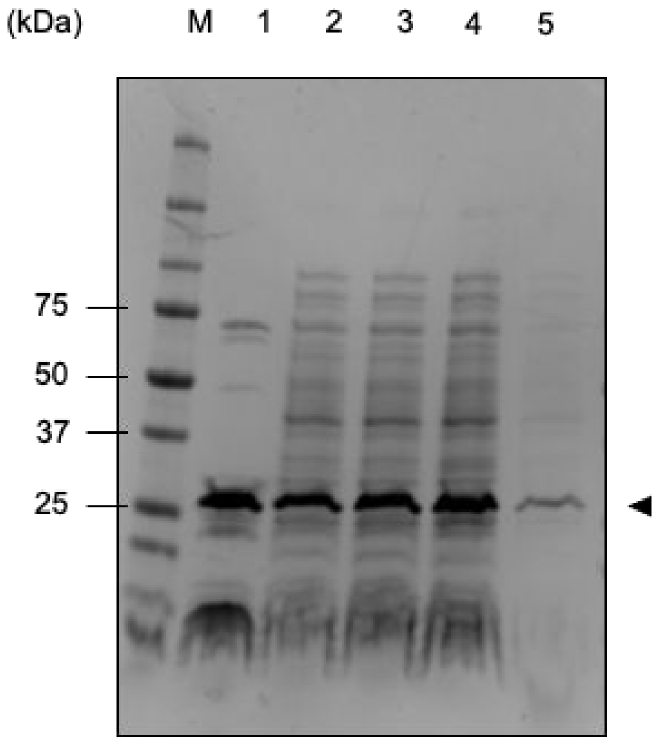

Figure S4. Expression and purification of ***B***-***M*** di-block construct (B^{RT}-E^{S}_{3}-M^{HR00C3_2}). SDS-PAGE analysis. Lane 1: IMAC eluate; lane 2: IMAC flow-through; lane 3: soluble lysate; lane 4: cell lysate; lane 5: intact cells; lane M: molecular weight marker.

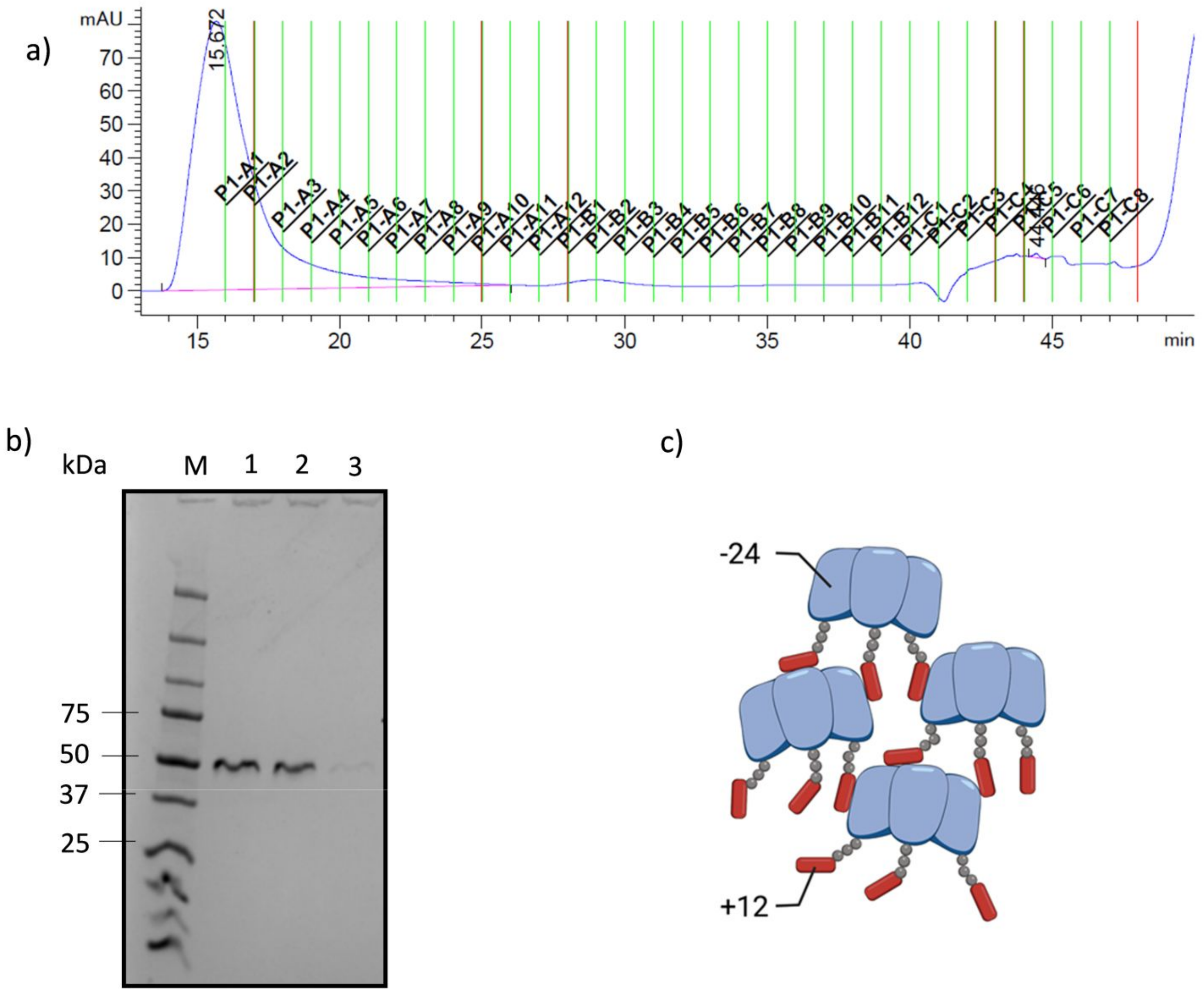

Figure S5 - Size exclusion chromatography of triblock construct ***B-M-E*** ($\boldsymbol{B}^{RT}$-$\boldsymbol{E}^{S}_{3}$-$\boldsymbol{M}^{HR00C3\_2}$-$\boldsymbol{E}^{S}_{40}$) at high salt. a) Chromatogram, absorbance at 280nm versus elution time. Superose 6 Increase 10/300 GL column, buffer is PBS + 500 mM NaCl pH 7.4, flow rate: 0.5 mL/min, fraction size: 0.5 mL. b) SDS-PAGE of fractions P1-A1 (lane 1), P1-A2 (lane 2) and P1-A3 (lane 3) c) Schematic illustration of possible electrostatic driving force for insolubility of ***B-M*** diblocks and salt-dependent bulk self-assembly of ***B-M-E*** triblock. Experimentally observed insolubility of the ***B-***

M diblock is likely due to electrostatic association of the positively charged binding blocks ***B*** (charge +12) with the negative charged trimer blocks ***M*** (charge -24). In the context of the trimer ***B-M-E*** we hypothesize the same interaction causes the experimentally observed salt-dependent reversible formation of higher order self-assemblies of ***B-M-E*** in solution
